# Systematic review and meta-analyses of studies analysing instructions to authors from 1987 to 2017

**DOI:** 10.1038/s41467-021-26027-y

**Published:** 2021-10-05

**Authors:** Mario Malički, Ana Jerončić, IJsbrand Jan Aalbersberg, Lex Bouter, Gerben ter Riet

**Affiliations:** 1 Urban Vitality Centre of Expertise, Amsterdam University of Applied Sciences, Amsterdam, The Netherlands; 2 Amsterdam UMC, University of Amsterdam, Department of Cardiology, Amsterdam, The Netherlands; 3 Department of Research in Biomedicine and Health, University of Split School of Medicine, Split, Croatia; 4 Elsevier, Amsterdam, The Netherlands; 5 Department of Philosophy, Faculty of Humanities, Vrije Universiteit, Amsterdam, The Netherlands; 6 Amsterdam UMC, Vrije Universiteit, Department of Epidemiology and Statistics, Amsterdam, The Netherlands

**Keywords:** Ethics, Funding, Publishing, Authorship, Journalism

## Abstract

To gain insight into changes of scholarly journals’ recommendations, we conducted a systematic review of studies that analysed journals’ Instructions to Authors (ItAs). We summarised results of 153 studies, and meta-analysed how often ItAs addressed: 1) authorship, 2) conflicts of interest, 3) data sharing, 4) ethics approval, 5) funding disclosure, and 6) International Committee of Medical Journal Editors’ Uniform Requirements for Manuscripts. For each topic we found large between-study heterogeneity. Here, we show six factors that explained most of that heterogeneity: 1) time (addressing of topics generally increased over time), 2) country (large differences found between countries), 3) database indexation (large differences found between databases), 4) impact factor (topics were more often addressed in highest than in lowest impact factor journals), 5) discipline (topics were more often addressed in Health Sciences than in other disciplines), and 6) sub-discipline (topics were more often addressed in general than in sub-disciplinary journals).

## Abstract (web summary)

Publishers’ policies have the capability to increase transparency in scholarly literature. Malički and colleagues carried out a systematic review of over 150 studies that have examined scholarly journals’ recommendations. They find that requirements in terms of authorship, conflict of interests, data sharing, funding disclosure or ethics approval declaration vary greatly over time, among journals and across disciplines.

## Introduction

Reporting of research differs between disciplines, and within journals of the same (sub)discipline, on the format and the structure of the manuscript, as well as on the level of detail with which the research is described^1–8^. Instructions to Authors (ItAs) are documents used by journals to describe specific requirements or recommendations authors should follow when reporting research and submitting their manuscript. Additionally, ItAs can describe the type of checks and review processes a journal employs in evaluating received submissions, and how authors or readers can address (suspected) irregularities in published papers^9–12^. ItAs can also be used to promote or raise awareness of standards^13–16^, and to depict methods aimed at reducing detrimental research practices, research waste, and the inability to replicate published research^17–19^.

Despite 350 years of scholarly publishing, and the existence of >43,000 scholarly journals^20^, research on ItAs, and on their evolution and change, is scarce. While it is common practice that journals update their ItAs, the breadth and the extent of changes to ItAs and their variability across disciplines have never been assessed, nor have the insights those changes or differences may provide about the history of scholarly publishing and the development of (best) reporting practices.

Therefore, we synthesised the findings of all studies that have analysed ItAs of more than one journal. After conducting a systematic review, we identified many factors associated with the percentage of ItAs addressing specific topics. Owing to discrepant findings across primary studies, we also conducted a series of meta-analysis to resolve those discrepancies. We focused on the ItAs’ recommendations regarding six research integrity topics: authorship, conflicts of interest, data sharing, ethics approval, funding disclosure, and International Committee of Medical Journal Editors Uniform Requirements for Manuscripts (URM).

In this work we summarise 153 studies that analysed ItAs from 1987 to 2017, and we showcase the timeline of ItA changes. We also provide evidence for six factors that explain a substantial part of the wide heterogeneity we found between journals’ coverage of the above-mentioned research integrity topics. Those six factors are: (1) time (year when the instructions were applicable), (2) country (in which the journals were published), (3) database (in which the journals were indexed), (4) impact factor, (5) scholarly discipline, and (6) sub-discipline.

## Results

### Study selection and characteristics

**Fig. 1:**
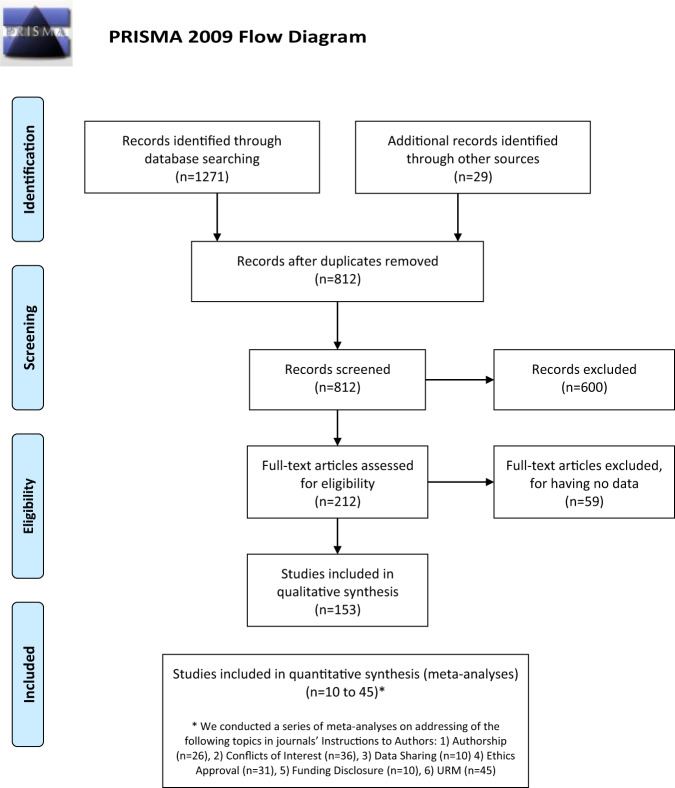
PRISMA flow diagram. After screening 812 records, we synthesized 153 studies analysing journals' Instructions to Authors (ItAs).

**Fig. 2:**
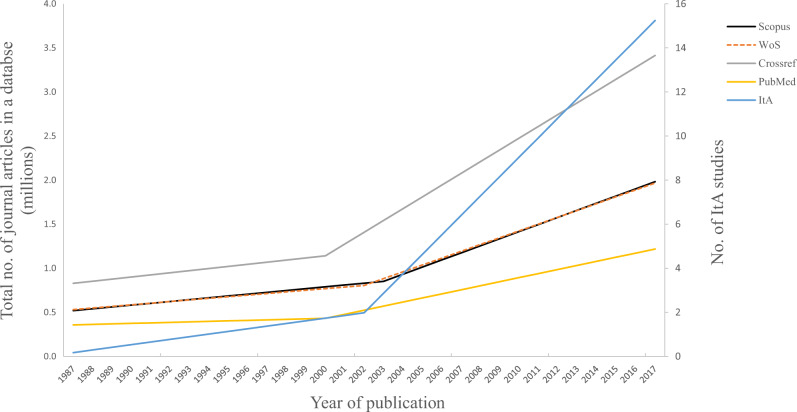
Growth of the number of publications analysing journals’ Instructions to Authors (ItAs). Growth of ItA studies is shown alongside that of journal articles in Crossref, PubMed, Scopus, and Web of Science. Prediction lines were determined by optimal spline regression models.

We identified 153 studies eligible for synthesis of results (Fig. 1 - PRISMA Flow Diagram)^21–173^. Study characteristics are shown in detail in Table 1. These studies were published over a thirty-year period (1987 to 2017) with an observed sharp rise in the number of studies following the year 2002 (and that growth was faster than for all articles published in that period, chi-squared tests, *p* < 0.0001 for all comparisons, Fig. 2).

**Table 1:** Characteristics of studies that analysed Instructions to Authors (ItAs) included in the systematic review, as well as those meta-analysed per specific topic.

	Systematic review (*n* = 153)	Meta-analyses					
		Authorship (*n* = 26)	Conflicts of interest (*n* = 36)	Data sharing (*n* = 10)	Ethics approval (*n* = 31)	Funding disclosure (*n* = 10)	ICMJE URM^a^ (*n* = 45)
Year of study publications (range)	1987–2017	1999–2017	1987–2017	1995–2016	1997–2017	1987–2016	1987–2017
Year of ItAs that were analysed (range)	1976–2016	1995–2015	1986–2015	1992–2015	1995–2015	1976–2015	1986–2016
No (%) of publications not listing the ItA year information	69 (45)	9 (35)	12 (33)	4 (40)	12 (39)	4 (36)	11 (24)
ItAs analysed per publication (median, range)	56 (3–1396)	57 (5–445)	54 (5–1396)	60 (5–850)	65 (4–208)	68 (6–216)	95 (4–747)
Discipline analysed (*n*, %)							
Arts and humanities	0 (0)	0 (0)	0 (0)	0 (0)	0 (0)	0 (0)	38 (84)
Health sciences	116 (76)	18 (69)	29 (81)	2 (20)	26 (84)	7 (64)	0 (0)
Life sciences	3 (2)	0 (0)	0 (0)	1 (10)	0 (0)	0 (0)	0 (0)
Physical sciences	3 (2)	0 (0)	0 (0)	0 (0)	0 (0)	0 (0)	0 (0)
Social sciences	7 (5)	1 (4)	0 (0)	0 (0)	0 (0)	0 (0)	1 (3)
Multiple	24 (16)	7 (27)	9 (19)	7 (70)	5 (16)	4 (36)	6 (13)
Countries/regions of journals analysed (*n*, %)							
Multiple	106 (69)	11 (42)	19 (53)	8 (80)	11 (35)	6 (55)	24 (53)
Brazil	12 (8)	1 (4)	4 (11)	0 (0)	8 (26)	1 (9)	5 (11)
India	6 (4)	3 (12)	2 (6)	0 (0)	6 (19)	1 (9)	4 (9)
China	5 (3)	1 (4)	1 (3)	0 (0)	0 (0)	0 (0)	1 (2)
Croatia	5 (3)	4 (15)	2 (6)	2 (20)	1 (3)	1 (9)	3 (7)
Spain	3 (2)	1 (4)	1 (3)	0 (0)	0 (0)	0 (0)	1 (2)
Mexico	2 (1)	0 (0)	1 (3)	0 (0)	0 (0)	0 (0)	0 (0)
South Korea	2 (1)	0 (0)	0 (0)	0 (0)	1 (3)	0 (0)	2 (4)
Other (1 per country)^b^	12 (8)	5 (20)	6 (17)	0 (0)	4 (13)	2 (18)	5 (11)
Journal selection methods (*n*, %)							
All journals within a database	88 (57)	23 (88)	28 (78)	6 (60)	25 (81)	8 (73)	32 (71)
No. of top journals within a database	35 (23)	0 (0)	2 (6)	3 (30)	1 (3)	0 (0)	9 (20)
Random sample of journals	1 (1)	0 (0)	0 (0)	0 (0)	0 (0)	0 (0)	0 (0)
All journals with an impact factor >10	1 (1)	0 (0)	0 (0)	0 (0)	0 (0)	0 (0)	0 (0)
All journals with an impact factor >2.63	1 (1)	0 (0)	0 (0)	0 (0)	0 (0)	0 (0)	0 (0)
Authors’ choice	9 (6)	1 (4)	3 (8)	1 (10)	1 (3)	1 (9)	0 (0)
A combination of methods	10 (7)	2 (8)	2 (6)	0 (0)	1 (3)	2 (18)	2 (4)
Selection method not listed	8 (5)	0 (0)	1 (3)	0 (0)	3 (10)	0 (0)	2 (4)
Analytic method (*n*, %)							
Not specified	94 (61)	12 (46)	19 (53)	5 (50)	21 (68)	5 (45)	20 (44)
Two independent coders	32 (21)	7 (27)	12 (33)	1 (10)	8 (26)	3 (27)	15 (33)
One coder	15 (10)	3 (11)	1 (3)	2 (20)	2 (6)	2 (18)	7 (16)
One author extracted the data, the other checked	6 (4)	2 (8)	1 (3)	1 (10)	0 (0)	0 (0)	1 (2)
One coder extracted data, the other checked, third checked a random sample	2 (1)	1 (4)	1 (3)	0 (0)	0 (0)	1 (9)	1 (2)
Two coders independently assessed a portion of the sample, then proceeded independently to extract from the remaining journals	2 (1)	0 (0)	1 (3)	0 (0)	0 (0)	0 (0)	0 (0)
One coder plus help of a text mining software	1 (1)	1 (4)	0 (0)	1 (10)	0 (0)	0 (0)	0 (0)
One author extracted sentences related to the topics analysed, then two proceeded to code the extracted sentences	1 (1)	0 (0)	1 (3)	0 (0)	0 (0)	0 (0)	1 (2)

^a^International Committee of Medical Journal Editors (ICMJE) Uniform Requirements for Manuscripts Submitted to Biomedical Journals (URM).

^b^Germany; Pakistan; Latin America and Caribbean; Spain and Latin American countries; Brazil, Mexico, Argentina, and Chile; Countries of Eastern and Southern Europe; Hungary and bordering countries; Japan; Cameroon; Taiwan; India and UK; Iran.

ItAs’ contents across these 153 studies were analysed for recommendations or requirements regarding more than a 100 different topics (extracted topics are available in our raw data file)^174^. We grouped those topics into 32 major topics (Table 2), of which the most commonly analysed were Research ethics (i.e., addressing of ethics approvals for conducting studies on humans or animals, *n* = 53, 34%), and Reporting guidelines (i.e., recommendations on items that should be reported for specific research studies, *n* = 51, 33%). The median number of major topics analysed per study was 2 (IQR 1–3). In almost half of the studies (*n* = 73, 48%) researchers also analysed if addressing of a topic was associated with one or more factors, with a total of 15 different factors explored across studies (Supplementary Table 1).

**Table 2:** Number of publications analysing a specific topic in journals’ Instructions to Authors (*N* = 153).

Topic	*n*	%	Topic	*n*	%
Research ethics	53	(34)	Journal’s scope	6	(3)
Reporting guidelines	51	(33)	Publication of supplementary materials	6	(3)
ICMJE URM	44	(28)	Abbreviations	5	(3)
Conflicts of interest	36	(23)	Acknowledgments	5	(3)
Authorship	35	(22)	Addressing of sex or ethnicity	5	(3)
Clinical trial registration	27	(17)	Editorial policies	4	(2)
Publication ethics	26	(16)	Journal financial disclosure	3	(1)
Manuscript formatting	20	(13)	Journal self-archiving policy	3	(1)
Accepted article type	15	(9)	Text legibility	2	(1)
Peer review	14	(9)	Registration of systematic reviews	2	(1)
Referencing	12	(7)	Submission format (e.g., email or print)	2	(1)
Copyright policy	11	(7)	Cover letter	1	(0)
Data sharing	11	(7)	Editorial freedom	1	(0)
Funding disclosure	10	(6)	Manuscript limitations	1	(0)
Statistics	7	(4)	Use of medical subject headings	1	(0)
Committee on publication ethics	6	(3)	Replication	1	(0)

### Narrative synthesis

We identified 12 different primary objectives authors listed for analysing ItAs (Supplementary Table 2), of which the most common were: (1) to determine if and how a specific topic was addressed in ItAs (*n* = 54, 35%); (2) to determine the reporting or citing of a specific topic in published papers and how the topic was addressed in ItAs (*n* = 51, 33%); (3) to recommend standards for a specific topic (*n* = 11, 7%).

Changes over time were analysed in 11 studies^23,25,26,31,39,43,59–61,67,75,85,91,103,115,134,141,162^, covering a time span from 3 to 11 years. Overall, these studies showed that topic coverage increased over time, most notably for: (a) depositing of DNA, amino acid sequence or protein structure data; (b) describing the peer review process; or c) recommending the use of Consolidated Standards of Reporting Trials (CONSORT) Guidelines (Supplementary Table 3).

Differences in reporting of topics in published papers between journals which covered those topics in their ItAs and journals that did not (or at the time when the topics were not addressed) were explored in 17 studies. These mostly showed that reporting is better in journals that covered the topics, albeit suboptimal (i.e., reported in <80% of articles, Supplementary Table 4). Suboptimal adherence to ItAs was also found in 12 studies which analysed if published papers adhered to requirements stated in ItAs (Supplementary Table 5).

### Series of meta-analyses

We conducted meta-analyses for prevalence of journals covering six research integrity topics: authorship, conflicts of interest, data sharing, ethics approval, funding disclosure, and URM. We chose these six topics due to our interest in research integrity, the project’s feasibility, and the number of studies that analysed these topics among the 153 identified studies (Table 1). Reported percentages of journals that covered these topics (with percentages being calculated by dividing the number of journals whose ItAs addressed a topic by the total number of journals whose ItAs were analysed in a particular study) for each individual study are available in our raw data file^174^. For each topic, we found large between-study heterogeneity (i.e., wide ranges of reported percentages, journal sample sizes, and journal selection methods); and in the series of meta-analyses we conducted (see Supplementary Section 2), we found strong effects of 6 factors that explained a substantial part of that heterogeneity, namely: (1) time, (2) country, (3) database indexation, (4) impact factor, (5) discipline, and (6) sub-discipline. However, as more than two-thirds of studies analysed ItAs of Health Sciences journals, these studies dominated the collective evidence. All confirmed effects in the meta-analyses, alongside associations that were reported in individual studies, but which could not be meta-analysed due to how data was reported, are presented in Table 3. Summary results for each factor are presented in subsections below. We chose not to report confidence intervals in the subsections below in order to avoid data overload and to allow for descriptive grouping across topics. However, all results per topic, with associated 95% CIs, are reported in the Supplementary Section 2. Additionally, as time trends were estimated using regression models, percentages reported below may differ from the percentages reported in individual studies.

**Table 3:** Factors associated with addressing of topics in journals’ Instructions to Authors (ItAs). Factors were confirmed by meta-regression or by demonstrating significant differences when data were obtained from up to three studies , calculated from data reported in individual studies  or reported as presented in individual studies, i.e., data reported in a way that did not allow calculation . All associations are presented only descriptively, with full details and 95% CIs available in the Supplementary Information. We used additional colouring to highlight , , and .




*The six disciplines we used in the study are: Arts & Humanities, Health, Life, Physical, Social, and Multidisciplinary Sciences. The specialties are sub-disciplines found within those areas (e.g., acoustics, botany, history, medicine, etc.).

Abbreviations and acronyms: Abridged Index Medicus (AIM), Chinese Medical Association Publishing House (CMPAH), Consolidated Standards of Reporting Trials (CONSORT), Directory of Open Access Journals (DOAJ), International Committee of Medical Journal Editors (ICMJE), Impact Factor (IF), Index Medicus (IM), Journal Citation Reports (JCR), U.S. National Library of Medicine bibliographic database (MEDLINE), PubMed Central (PMC), World Association of Medical Editors (WAME).

### Time

**Fig. 3:**
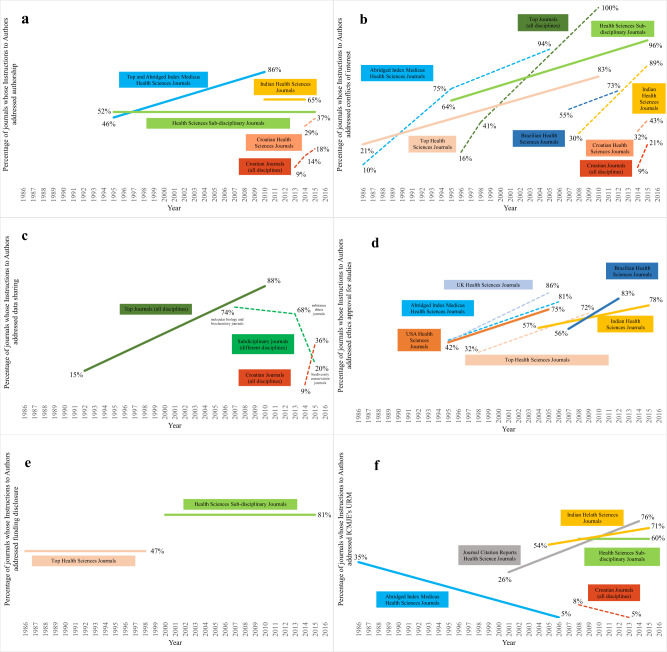
Changes over time in addressing publication ethics in journals’ Instructions to Authors. For ease of comparison all panels are on the same scale, with vertical axes based on a logit scale and percentages added as reference points. Panels represent changes over time for authorship (**a**), conflicts of interest (**b**), data sharing (**c**), ethics approval (**d**), funding (**e**), and International Committee of Medical Journal Editors Uniform Requirements for Manuscripts (**f**). Full lines represent trends obtained through regression models, while dash lines represent percentages reported in up to three studies.

We found large differences between 1986 and 2016 in percentages of ItAs addressing the six above-mentioned research integrity topics. Overall topic coverage generally increased over time. For example, while in 1995, ~40% of top or Abridged Index Medicus Health Sciences journals addressed authorship and ethics approval, by 2005, >70% of those journals did so (Fig. 3 and Supplementary Information). In the same period, a similar increase was found in UK and USA Health Sciences journals for ethics approval, while Indian and Brazilian Health Sciences journals experienced an increase a decade later (Indian journals, from 57% in 2004 to 78% in 2015, and Brazilian journals, from 56% in 2007 to 83% in 2012).

An increase over time, however, was not ubiquitous for all topics, (sub)disciplines or countries. In Health Sciences sub-disciplinary journals, addressing of conflicts of interest increased from 57% in 1995 to 87% in 2015, but those journals showed no increase after 2000 for URM (60%), authorship (65%), or funding disclosure (81%).

We also observed a decrease in addressing of URM in Abridged Index Medicus Health Sciences journals, from 35% in 1986 to 5% in 2006.

Data on changes over time for non-Health Sciences journals was scarce. Top journals (of all disciplines) showed an increase for data sharing (from 15% in 1992), and for conflicts of interest (from 16% in 1997), to >85% for both topics by the year 2010 (Fig. 3). Additionally, we found an indication of an increase over time for Croatian journals (from all disciplines) for authorship, conflicts of interest, and data sharing from ~9% in 2013 or 2014 to >20% in 2015 (Supplementary Information).

### Country

We found large differences in addressing of topics between countries. Almost always, topic coverage was lower in journals from a single country than among top or general Health Sciences journals. For example, while in 2010 ~83% of top journals (of all disciplines) addressed conflicts of interest, in 2014, 89% of Indian Health Sciences journals did so, and only 9% of Croatian journals. Similarly, while in 2014 almost 90% of top Health Sciences journals addressed authorship, 86% of Chinese journals did so, 70% of Indian, and 29% of Croatian journals.

Among Health Sciences journals, addressing of conflicts of interest (89% in 2014), funding disclosure (70% in 2008), and URM (75% in 2014) was most prevalent in Indian journals, ethics approval (86% in 2005) in UK journals, and authorship (86% in 2014) in Chinese journals. Chinese journals, however, had the lowest coverage of URM (7% in 2011).

Country-specific data for journals of all disciplines was only available for Cameroon in 2009, and Croatia for periods 2012 to 2015, with coverage of all topics found in <37% of journals (Supplementary Information).

### Journal indexation

Journal indexation was associated with covering of all topics except data sharing (for which no studies provided data for journals from different databases). For example, in 1986, higher percentage of top Health Sciences journals addressed funding disclosure than did Abridged Index Medicus journals (47% vs. 22%, respectively). That situation was reversed for ethics approval 20 years later: with 81% of Abridged Index Medicus journals in 2006 vs. 71% of top Health Sciences journals in 2009 (Supplementary Information). Additionally, while Health Sciences journals indexed in Journal Citation Reports (JCR) showed an increase in URM coverage from 2001 (22%) to 2014 (77%), Abridged Index Medicus Health Sciences journals showed a decrease between 1986 (37%) and 2006 (5%).

For Health Sciences sub-disciplinary journals, almost no differences were found between journals indexed in Directory of Open Access Journals (DOAJ), Index Medicus (IM) or JCR between 2008 and 2016 for URM (60%), however paediatrics journals index in JCR addressed conflicts of interest (78%) more often than DOAJ indexed journals (63%).

### Impact factor

We found weak indications that the coverage of authorship, conflicts of interest, and URM was associated with impact factor for Health Sciences sub-disciplinary journals (no data was available for other disciplines, or even for general Health Sciences journals). Specifically, for conflicts of interest, higher coverage was found for IF ≥ 3 journals (85%) compared to journals with IF < 1 (72%) between 2008 and 2016, as well as for URM (74% vs. 50%). For authorship, higher coverage was found in 2010 for journals with IF values of 1 to 2 (61%) than for those with IF < 1 (26%). Single studies, and studies reporting correlation analyses with IF yielded inconclusive evidence (see additional analyses below and the Supplementary Information).

### Discipline

We found large disciplinary differences for all topics, with Health Science journals more often addressing all six research integrity topics (e.g., in 2010 in Web of Science, 59% of Health Sciences journals addressed authorship vs. 7% of Arts & Humanities journals).

However, only 1–3 studies per topic reported disciplinary data, and those were either based on data from 2010 onwards or belonged to country or region-specific disciplinary journals (Croatia, Spain and Latin America, or Spain and the Caribbean, Supplementary Information).

### Sub-discipline

Generally, topics were less often addressed in Health Sciences sub-disciplinary than in top or general Health Sciences journals. For authorship, funding disclosure, and URM there were almost no sub-disciplinary differences (52% for authorship between 1995 and 2015, 81% for funding disclosure between 2000 and 2015, and 60% for URM between 2008 and 2016, Fig. 3). However, we found large differences for ethics approval and conflicts of interest between sub-disciplinary journals of different disciplines (Table 3 and Supplementary Information).

### Additional analyses

Individual studies explored the association of seven additional factors with addressing of research integrity topics in ItAs, but again only for Health Sciences journals. The explored factors were language, publishers, endorsement of International Committee of Medical Journal Editors (ICMJE), endorsement of Consolidated Standards of Reporting Trials (CONSORT), membership in World Association of Medical Editors (WAME), in South Korean Medical Association (SHMA), or Chinese Medical Association Publishing House (CMPAH).

Except for language, for which one study in 2012 reported Iranian journals publishing in English covering authorship and conflicts of interest more often than journals publishing in Farsi^112^, all other explored factors were found to be associated with some topics, while not with others (e.g., more ICMJE endorsing journals addressed ethics approval and URM, but not authorship or conflicts of interest when compared to non-endorsing journals).

Similarly, studies that reported on associations with impact factor values (but without providing data that could be used in meta-analyses) also reported conflicting results (Table 3 and Supplementary Information).

## Discussion

Our systematic review identified 153 studies that analysed journals’ Instruction to Authors (ItAs) and 12 main objectives listed for conducting these studies, of which the most common were to determine whether journals had embraced particular policies or expert recommendations (e.g., reporting of ethics approval), and if papers published in those journals adhere to the journals’ requirements. Such interest in journals’ ItAs might reflect the attitudes and expectations of researchers that journals and editors should promote best scholarly practices and ensure the highest quality of papers they publish^175,176^. We also found an increase in the number of studies analysing ItAs after 2002, which might reflect the switch to online publishing and the ease of obtaining digital instead of printed versions of ItAs and published papers, as well as the rise in numbers of meta-research studies over the last two decades^177^.

Although there are indications, both in our study, and in the recently published scoping review^178^, that recommendations or requirements stated in ItAs are associated with better study reporting, more studies are needed to identify the best methods for ensuring authors’ compliance with ItA’s, as well as for conducting editors’ and reviewers’ checks of that compliance. Future studies could also investigate how often and how well ItAs are read by the authors, and the effect ItAs might have on raising awareness of the topics they address.

Our series of meta-analyses on six research integrity topics (authorship, conflicts of interest, data sharing, ethics approval, funding disclosure, and URM) found six factors that were associated with the addressing of those topics in journals’ ItAs: time, country, database indexation, impact factor, discipline, and sub-discipline.

The overall increase in the number of journals addressing these topics over the last 30 years may be a results of several factors that include: the improvement of scholarly methods, the progress in teaching of those methods and standards of reporting^179,180^, the rise in (inter)national regulations, especially regulations on obtaining ethics approval for studies^181^, as well as increased attention to research integrity. The fact that most studies analysed ItAs of Health Sciences journals, and that Health Sciences journals covered these topics more frequently than journals of other disciplines, is most likely due to the strict regulations of experimentation on humans and animals, as well as increasing calls in Health Sciences for studies on editorial processes, peer review, research integrity, research waste and replication studies^18,175,182–184^. This could also indicate that Health Sciences journals might be leading the way in reporting practices for journals in other disciplines.

However, while the above findings paint an overall positive picture of the changes in ItAs over the last 30 years for these six research integrity topics, we found many exceptions to those trends. For example, ~52% of Health Sciences sub-disciplinary journals addressed authorship between 1995 and 2015; and there were also large differences in addressing of research integrity topics between countries (e.g., 14% of Croatian journals addressed authorship in 2014 vs. 70% of Indian journals). This indicates that many journals still lag behind recommending, requiring or implementing best reporting practices, which was also confirmed in our recent cross-sectional analysis of 19 transparency in reporting and research integrity topics across disciplines^12^.

Furthermore, while we identified 153 studies that analysed the content of ItAs, the fact that most analysed only specific (sub)samples of journals and looked at addressing of only one or two topics within them, highlights the need for a comprehensive database that would allow authors or other stakeholders to compare journals based on their ItA requirements or recommendations (i.e., akin to SHERPA/RoMEO for listing of journal open-access and self-archiving policies^185^, the Platform for Responsible Editorial Policies^186^ or TOP Factor indicators^187^). Such a database could also include indicators of adherence of publications with the journal’s requirements. Thus, it could function like Trials Tracker^188^ for monitoring the compliance with EU or FDA regulations on timely posting clinical trial results. It could also enable mapping of changes in ItAs and journals’ policies over time, while also providing a quality indicator or reputation safeguard for the journals.

Note that in the meta-analyses we conducted, we found associations for all six factors that we had data for, and that our narrative review showed indications of associations with an additional nine factors. And yet, many other factors potentially associated with the ItAs’ contents, like the influence of specific editors or (large) publishers, changes made when a journal reaches a certain level of prestige^189^, or (high profile) misconduct or legal cases, have not been explicitly explored in these studies (the exception being one study which showed differences between open-access publishing houses, professional organisation publishers, and other publishers for addressing of conflict of interest, but not for URM)^90^.

Finally, both the meta-analyses we conducted and the percentages reported in individual studies on topics we did not meta-analyse, show that different topics follow different patterns, i.e., one topic being addressed in ItAs does not mean another one will be addressed too, nor that its coverage follows the same time trend, and so we warn against generalisation of the patterns we found for the six research integrity topics to other topics.

The strength of our study lies in the fact that we used the systematic review methodology to gather all studies analysing contents of ItAs of more than one journal, rather than focusing on a specific topic(s) or outcomes. But it also has several limitations. First, following our interests and project feasibility, we chose to meta-analyse only topics related to research integrity. Even though these topics were also among the most researched in the studies that analysed ItAs, further research should explore time trends and factors associated with addressing of other topics. Second, previous studies have shown that some enforced practices are not always listed in ItAs^190,191^, while others, including studies listed in our narrative review, that listed practices are not always enforced^111,192^, and finally, that some topics are reported in published papers even if not addressed in ItAs^105^. Afterall, ItAs are not meant to preclude authors for adhering to better reporting, and some authors are likely to go beyond (minimum) requirements imposed by the journals. Third, the association of countries, language and disciplines on reporting of research integrity topics have been demonstrated on a very small number of studies (1–5), of which the strongest indications come from two studies by the same author who looked at the ItAs of Croatian journals^143,144^. So further research into these associations is warranted. Finally, we have summarised information on addressing of topics in ItAs in a binary way (whether or not they were addressed), not on how each individual topic was addressed (e.g., the fact that authorship was addressed in ItAs, does not mean that all journals had the same requirements for authorship, nor that they addressed the number, order of authors, or the practice of shared authorship).

In conclusion, while our findings provide evidence that addressing of these six research integrity topics in journals’ ItAs had increased over time, they also showed that many (sub)discipline and regional journals still lag behind in providing such guidance to authors. If publishers, editors and journals want to increase and safeguard the quality and transparency of reporting, they could benefit from updating and implementing policies that reflect and strengthen the integrity of research.

## Methods

We adhered to the Preferred Reporting Items for Systematic Reviews and Meta-Analyses (PRISMA) guidelines^193^.

### Protocol and registration

We could not preregister the study in PROSPERO as it did not include any health-related outcome; however, our projects’ data repository site contains information on the conception of the study, as well as all the data and notes associated with it^174^.

### Eligibility criteria

We searched and included all studies that analysed ItAs of more than one journal, irrespective of the topic(s) ItAs were analysed for.

### Information sources

We conducted the search on 1 May 2017 in three databases: MEDLINE (through Ovid interface), Scopus, and Web of Science (WoS) with no language or time restrictions. We also searched Google Scholar with the query -allintitle: instructions authors) -, and references of all included studies.

### Search

The full search strategy for all three databases is available on our project’s data repository site^174^.

### Study selection

We exported the search results of the three databases into Rayyan software^194^, where manual deduplication was done by MM. Abstracts were assessed independently by MM and AJ to remove irrelevant studies. Disagreed upon studies were obtained in full (*n* = 25). Additional publications were identified through Google Scholar, through searching of references of selected studies, or through authors’ awareness of published studies. Full texts of publications were checked by both assessors to confirm the eligibility criteria, and extract topics that were analysed in ItAs and the percentages of journals addressing those topics calculated based on all journals analysed in those studies.

### Data collection process and data items

For each included study, MM extracted the following data in Excel: (1) number of journals whose ItAs were analysed within a study, (2) sampling method for choosing these journals, (3) discipline to which the analysed journals belonged to (reported disciplines were reclassified to fit the following categories: Arts & Humanities, Health Sciences, Life Sciences, Physical Sciences, Social Sciences, and Multidisciplinary Sciences), (4) sub-discipline to which the journals belonged to (as specified in the respective studies, e.g., dental medicine for Health Sciences), (5) countries or territories in which the journals were published, (6) year when the journals’ ItAs were accessed/analysed, (7) topic(s) that were analysed, (8) number and percentage of journals addressing a topic (out of the total number of journals whose ItAs were analysed in a study), (9) method(s) of analysing the ItAs (e.g., one or more researchers reading the ItAs), (10) factors explored for possible association with addressing a particular topic (e.g., journal’s impact factor, indexed database, or publisher), and (11) objectives or hypotheses listed as reasons for conducting the study. The (names of) databases in which the journals were indexed were extracted as reported in original studies, we did not assign database indexation to studies in which they were not reported as only a third of studies reported a full list of journals they analysed (*n* = 53, 35%, see below). For all included studies AJ then checked if the data extraction was done correctly.

Additionally, the following variables were also extracted, but were not included in the results synthesis: (1) if the authors also surveyed editors about questions relating to the submission process, (2) if the studies included a detailed list of journals whose ItAs were analysed.

The data extraction process revealed that >100 different topics were analysed across the studies, and so we grouped them into major topic variable, while we also kept a record when studies included other (sub)topics for which we did not extract the data (all recorded sub-topics are available at our project’s data repository site)^174^. To clarify, we list here two examples of sub-topics: (1) for the topic Reporting Guidelines, the sub-topics were: mentioning of different specific reporting guidelines; (2) for the topic Case Reports (mentioning of publishing case report studies in the journal), the sub-topics were: maximum word count allowed or requiring a structured abstract.

Finally, on 4 December 2020, we extracted the number of journal articles in PubMed, WoS, Scopus and Crossref. Searches and extracted numbers are available on our project’s data repository site^174^.

### Synthesis of results and additional analysis

No meta-analyses were predefined at the study conception stage, as the number of topics analysed and the factors explored as determinants of journals addressing a topic could only be assessed after conducting the systematic review.

To study the time trend in the number of studies analysing ItAs and the number of journal publications in PubMed, WoS, Scopus and Crossref, we used a spline regression model that was fitted to the data using nonlinear regression within the SPSS v24 software (IBM, Chicago, IL, USA). Comparisons of number of published studies analysing ItAs published ≤2002 vs. >2002, with number of articles published in PubMed, WoS, Scopus, or Crossref in the same period were conducted with a series of chi-squared tests.

Meta-analyses of percentages of journals addressing a particular research integrity topic in ItAs were performed using Comprehensive Meta-analysis Software (CMA) version 3 (Biostat Inc., Englewood, USA), which pools percentages using the logit transformation method. The percentages with their 95% confidence intervals (CI) were calculated within CMA software from the number of journals addressing a topic out of the total number of journals analysed in a particular study. If percentages reported in a study were 0% or 100%, CMA introduced a continuity correction to avoid including studies with standard errors of zero.

Random-effects models were used to estimate summary percentages. However, because estimation of random-effects models with few studies have been shown to be unreliable^195^, if fewer than five studies were included in a meta-analysis we applied a fixed-effect model. Statistical heterogeneity of studies was estimated with both Cochran’s Q test, and Higgins’s *I*^2^ test statistic. In case of considerable heterogeneity, we did not pool the data, as listing a summary percentage could be misleading. In those cases, to explain the heterogeneity, we conducted mixed-effects subgroup analyses (for categorical factors such as countries, disciplines, or impact factor categories reported in the studies) or random-effects meta-regressions based on the DerSimonian and Laird method, which doesn’t assume that effect sizes are normally distributed (for the factors time, impact factor values, discipline, or indexed database). We also searched for sources of heterogeneity if percentages were dispersed throughout the 0 to 100% interval, but due to high uncertainty 95% CIs largely overlapped). If the number of studies was too small to perform meta-regression and a factor was numerical (i.e., for *n* < 4 when the factor time was assessed), we decided that a factor significantly affects the percentages if we could show: (a) consistency in direction of change in percentages with growing values of the factor, and (b) significant differences between percentages assigned to neighbouring values of a factor. Differences in percentages reported in two studies, together with associated 95% CIs for the difference, as well as *p*-values for statistical test of differences, were estimated by Exploratory Software for Confidence Intervals (ECSI) software^196^. Pseudo-R^2^ index was used to quantify the proportion of variance explained by a factor.

A *p*-value of <0.05 was considered to indicate statistical significance. However, when analyses were underpowered (e.g., for comparison of two estimates with one being made on a sample of five journals), we also stated if a result was significant at the 0.1 level. All the tests were two-sided.

### Risk of bias

We are aware of no tools that measure the risk of bias of studies whose units of analysis are ItAs. As the methodology of these studies involves selecting journals for analysis, obtaining ItAs from printed volumes or downloading them from journal’s websites, and extracting data on topics addressed in ItAs by reading them (by one or more researchers) or using qualitative text analysis software, we included all eligible studies in synthesis of results and meta-analyses. We, however, did provide notes, in both Table 3 and the Supplementary Section 2, about methodological factors, which could have an effect on the validity and reliability of reported estimates. In regards to selection bias, none of the studies used probability sampling that would cover all disciplines and a wide range of journal (citation) influences or indexing databases. Rather, most studies analysed ItAs of Health Sciences journals (*n* = 116, 76%), and sampled only journals that were indexed in the Journal Citation Reports database (*n* = 55, 47%). In regards to reporting bias, studies often omitted explaining their methods of analysing ItAs (*n* = 94, 61%), or listing the year of ItAs that were analysed (*n* = 69, 45%, Table 1).

## Supplementary information

Supplementary Information

Peer Review File

## Data Availability

The data generated in this study have been deposited in the Mendeley repository with the identifier: https://data.mendeley.com/datasets/53cskwwpdn/5^174^.
