# Supplementary material for: Systematic review and meta-analyses of studies analysing instructions to authors from 1987 to 2017
Source: Nat Commun. 2021 Oct 5;12:5840. doi: 10.1038/s41467-021-26027-y (PMC8492806; doi:10.1038/s41467-021-26027-y)
Supplement: Supplementary file 1 — Supplementary Information [file 41467_2021_26027_MOESM1_ESM.pdf]

Supplementary Information

to ***Systematic Review and Meta-Analyses of Studies Analysing Instructions to Authors from 1987 to 2017*** *by Mario Malički, Ana Jerončić, IJsbrand Jan Aalbersberg, Lex Bouter, and Gerben ter Riet.*

This supplement is divided in two sections: Section 1 refers to tables and figures referenced in the systematic review results and narrative summary; while Section 2 includes detailed information on the series of meta-analyses we conducted. Table of contents is presented below.

Contents

Section 1. Systematic Review 2
- Supplementary Table 1. 2
- Supplementary Table 2. 2
- Supplementary Table 3. 3
- Supplementary Table 4. 5
- Supplementary Table 5. 7

Section 2. Series of Meta Analyses 8
- Authorship 8
- Conflicts of interest 13
- Data Sharing 19
- Ethics approval 23
- Funding disclosure 27
- Uniform Requirements for Manuscripts (URM) 29

References: 34

Section 1. Systematic Review

Supplementary Table 1. Factors explored as possible determinants of topics being addressed in journals' Instructions to Authors (N=153).

Factor	n	%
Impact Factor	30	(19)
Subdiscipline (e.g., Cardiology vs General Medicine)	26	(16)
Region/Country	14	(9)
ICMJE endorsement	9	(5)
Database indexation (e.g., AIM vs non-AIM)	8	(5)
Publisher	7	(4)
Discipline (e.g., Humanities vs Health)	5	(3)
Publishing model (e.g., Open Access vs non-OA)	4	(2)
Publication Language	2	(1)
Committee on Publication Ethics Membership	1	(0)
Qualis Classification	1	(0)
Journal published by or in association with any medical association	1	(0)
World Association of Medical Editors Membership	1	(0)
Journal's Age	1	(0)
Korean Medical Association Membership	1	(0)

Abbreviations: AIM - ICMJE - International Committee of Medical Journal Editors, Abridged Index Medicus, Quails - Brazilian government classification system of scientific productions

Supplementary Table 2. Primary objectives listed in studies (N=153) analysing journals' Instructions to Authors (ItAs).

Objective	n	%
To determine if and how topics are addressed in ItAs	54	(35)
To determine the reporting or citation of topics in manuscripts and addressing of topics in ItAs	51	(33)
To recommend standards for topics	11	(7)
To determine factors associated with differences in ItAs	9	(6)
To raise awareness of a topic(s) and its mention in ItAs	8	(5)
To determine changes in ItAs over time	7	(4)
To determine addressing of a topic(s) in ItAs and policy documents	5	(3)
To improve the quality of journals within a country	4	(3)
To determine if ItAs can be a proxy for journal quality and library subscription decisions	2	(1)
To determine if there is a standard way of addressing a topic	1	(0)
To determine how authors deal with differences in ItAs between journals	1	(0)
To provide guidance on writing and updating ItAs	1	(0)

Supplementary Table 3. Cohort studies that analysed changes in journals' Instructions to Authors (ItAs) over time.

Ref.	Discipline	No. of journals analysed	Journal selection method	Topic analysed within instructions to authors	Percentage of journals' instructions mentioning the topic in a given year			Change
					**1976**		**1985/1986**	
23	Health Sciences	93	All journals from *Abridged Index Medicus*	Peer Review	23		68	↑
				Ethics	28		44	↑
				Journal's Scope	19		44	↑
				Average page length of instructions	1.27		1.5	↑
					**1982***	**1992***	**2001***	
38	Life Sciences	23	Journals with most papers containing the keyword "GenBank"	Requiring deposit of sequence data into a genomic and proteomic database	0	78	100	↑
					**1988-1991**		**1994/1995**	
25, 31	Health Sciences	50,60 (38) ‡	CENDIS database	Type of article accepted	82		63	↓
				Symbols and abbreviations	34		52	↑
				Abstract word limit	90		93	↑
				Structure of the abstract	86		73	↓
				Statistics	12		12	=
					**1995**		**2005**	
75	Health Sciences	103	All journals from *Abridged Index Medicus*	Ethics Approval	42		76	↑
				ICMJE URM	17		4	↓
				Declaration of Helsinki	3		4	↑
				Informed Consent	9		1	↓
				Conflict of Interest	75		94	↑
				Definition of Authorship	40		72	↑
				Prohibiting Simultaneous Submission	90		96	↑
				Prohibiting Duplicate Publication	88		95	↑
					**1995**		**2006**	
26, 60	Health Sciences	102/101†	All journals from *Abridged Index Medicus*	Ethics Approval	47		83	↑
				Guidelines for research on humans	76		92	↓
				Declaration of Helsinki	3		3	=
				Informed Consent	10		1	↓
				ICMJE URM	15		5	↓
					**2000**	**2005**	**2010**	
91	Health Sciences	9	JCR General Medicine Category	Acknowledgments	33	44	44	↑
				Authorship	55	55	55	=
				Conflict of Interest	66	77	77	↑
				Declaring Funding	88	88	66	↓
				ICMJE Authorship	33	33	33	=
				Ethics Approval	44	44	55	↑
				Reporting Guidelines	11	44	44	↑
				Trial Registration	0	11	33	↑
					**2000**		**2005/2006**	

39, 59	Social Sciences	34/31†	Magazines for Libraries list of Library and information journals	Web site persistence (i.e., DOI)	0		10	↑
				Referencing electronic resources	18		39	↑
43, 61, 162	Health Sciences	167/165/ 168†	JCR's top 5 from 33 med. spec. and top 15 general and internal medicine		**2003**	**2007**	**2014**	
				CONSORT	22	38	63	↑
				ICMJE URM	43	42	77	↑
				Trial Registration	NR	37	63	↑
67, 115	Health Sciences	65/67†	Indian medical journals identified in 5 bibliographic databases		**2004/2005**		**2007/2008**	
				CONSORT	31		33	↑
				ICMJE URM	58		52	↓
				Ethics Approval	55		70	↑
				Trial Registration	2		16	↑
85,134	All sciences	163, 289†	All journals from the central portal of Croatian scientific journals		**2008**		**2013**	
				Policy for m. submissions by editors	0		1	↑
				ICMJE URM	8		5	↓
				COPE	1		4	↑
141, 103	Health Sciences	134	JCR Surgery Category		**2011**		**2014**	
				CONSORT	30		42	↑
				PRISMA/QUORUM	10		19	↑
				Trial Registration	33		42	↑

*The study reported mentioning of data deposit for each year from 1982 to 2001, we divided it into these 3 time periods to match the other studies.

† Some of the journals ceased publication over time or split into two journals.

‡The study used the same methods of selecting journals, all indexed in the national database, while 50 journals were indexed when the first study was conducted, 60 were indexed when the follow up was done, of which 38 overlap, however data for just the 38 were not available. Additionally, the study also reported details of manuscript formatting that are not included in the table.

Acronyms: ICMJE URM - The International Committee of Medical Journal Editors Uniform Requirement for Manuscripts, JCR – Journal Citation Reports, CONSORT - Consolidated Standards of Reporting Trials, COPE - Committee on Publication Ethics, PRISMA/QUORUM - Preferred Reporting Items for Systematic Reviews and Meta-Analyses/Quality of Reporting of Meta-analyses, DOI - Digital Object Identifier, CENDIS – Centro National de Informacion y Documentacion en Salud – Mexican National Center for Health Information and Documentation.

Supplementary Table 4. Studies that analysed differences in manuscripts in relation to the content of Instructions to Authors (ItAs).

Ref.	Discipline	No. of journals (no. of. manuscripts) analysed		Topic(s) (evaluation method, min – max score)	Reported Means, N or % for Journals		P (value or range)
		Addressing the topic(s)	Not addressing the topic(s)		Addressing the topic(s)	Not addressing the topic(s)	
142	Health Sciences	7 (171)	30 (232)	CONSORT checklist items score (1-84)	M=29.47	M=25.57	NR
				JADAD score (1-5)	M=2.53	M=1.97	NR
67	Health Sciences	20 (31)	45 (120)	CONSORT items score (1-13)	M=5.55	M=4.93	0.15
				JADAD score (1-5)	M=2.03	M=1.76	0.21
128	Health Sciences	5 (29)	42 (141)	Race and ethnicity features	1 (3%) to 26 (90%) articles	NR	0.000 to 0.521
				Limitations	13 (45%) articles	NR	0.008
				Hypothesis	10 (34%) articles	NR	0.730
100*	Health Sciences	2 (327)	2 (437)	CONSORT for Abstracts (0-9)	Pre-implementation M=2.56	Post-implementation M=5.41	0.0037
120*	Health Sciences	NR	NR	CONSORT-NPT item Adherence	Pre-implementation 31%	Post-implementation 34%	0.4592
				STRICTA item Adherence	Pre-implementation 58%	Post-implementation 79%	0.0042
63	Health Sciences	4 (8)	44 (34)	OQAQ Methodologic Quality (1-9)	M=8.5	M=4.6	<0.001
				OQAQ Question 10 (1-7)	M=5.0	M=2.4	<0.001
				OQAQ Methodologic Errors (%)‡	1 (13%) Major e. 7 (87%) Minor e.	26 (76%) Major e. 8 (24%) Minor e.	<0.001
				Adherence to QUOROM (1-18)	M=16.1	M=9.3	<0.001
151	Health Sciences	7 (NR)	3 (NR)	Adherence to ethics approval and informed consent	OR 0.32 for journals addressing the topics (95% CI 0.15-1). "68% articles are less likely to report topics when the journals have not addressed them"		0.05
159	Health Sciences	18 (18)	18 (18)	STREGA (% for 22 items)	63%	56%	0.04
168	Health Sciences	7 (13)	21 (40)	Adherence to CONSORT-PRO	3 predictors of adherence score: 'citing CONSORTPRO', 'journal endorsing CONSORT-PRO' and 'dedicated PRO paper' (R^2 = 0.48)		<0.001
130	Health Sciences	8 (8)	4 (4)	No. of journals using textual alternatives to figures on article pages	0	3	0.0182†
				No. of journals using alternatives to figures in PDF(s)	8	3	0.333
110*	Health Sciences	9 (15)	9 (30)	PRISMA Adherence (%)	Pre-implementation 83.1%	Post-implementation 90.1%	0.003
				AMSTAR Adherence (%)	Pre-implementation 74.6%	Post-implementation 85%	0.002
		9 (30)	61 (30)	PRISMA Adherence (%)	90.6%	85.3%	0.003
				AMSTAR Adherence (%)	85.3%	76.9%	0.016
160	Health Sciences	5 (69)	5 (151)	Reporting of confounding	Md=4	Md=4	0.33

				based on STROBE (1-8)			
111	Health Sciences	Not reported	Not reported	Trial Registration	No difference in the proportion of registered trials between journals that required trial registration and those that did not.		0.073
171	Health Sciences	30 (37)	77 (37)	PRISMA (adherence %)	64.9%	73.0%	>0.05
				AMSTAR (adherence %)	18.2%	18.2%	>0.05
106	Social Sciences	26 (737)	46 (433)	Supplementary Materials Guidance	All journals published manuscripts with supplementary materials.		>0.05†
129	Physical Sciences	0-6 (NR)	5-11 (NR)	Image accessibility issues	Image accessibility issues were mostly inconsistent with ItAs		NR
83	Health Sciences	6 (149)	44 (351)	Data Sharing	208 (59%) of 351 articles did not fully adhere to data availability instructions of the journals they were published in. None of 149 articles of journals that had no policies shared primary data.		NR

*Measured adherence before and after introduction of guideline endorsement at a journal
† Not reported in the article, calculated using Fisher exact test
‡ Studies were deemed to contain major methodological errors if the overall *Overview Quality Assessment Questionnaire* score (range 1 to 10) was less than or equal to 3, or minor if the was greater than or equal to 4.
Acronyms: AMSTAR - A Measurement Tool to Assess the Methodological Quality of Systematic Reviews, CONSORT - The Consolidated Standards of Reporting Trials Statement, CONSSORT-NPT– CONSORT for Non-Pharmacological Trials, CONSORT-PRO – CONSORT Patient Reported Outcomes statement, JADAD Scale - 5-point scale for evaluating the quality of randomized trials, NR – Not Reported, OQAQ - Overview Quality Assessment Questionnaire, QUOROM - Quality of Reporting of Meta-Analyses guidelines, STREGA - Strengthening the Reporting of Genetic Association studies, STROBE - Strengthening the Reporting of Observational studies in Epidemiology

Supplementary Table 5. Studies that analysed if published articles adhere to requirements stated in journals' Instructions to Authors (ItAs).

Ref.	Discipline	No. of journals (manuscripts)	Topic(s)	Results (no. and % of manuscripts addressing the topic or outcomes reported)	Optimal Adherence (> 80% of Manuscripts)
132	Health Sciences	3 (50)	CONSORT Flow Diagram	16 (32)	**No**
			Trial Registration	0 (0)	**No**
107	Social Sciences	11 (239)	Reporting of RCTs (7 different guidelines)	Trials reported a mean of 42% of reporting standards	**No**
154	Health Sciences	8 (90)	CONSORT item Adherence	Randomization 70%, Allocation 45%, Blinding 52%, Flowchart 91%	**For some items**
74	Health Sciences	4 (534)	Ethics Approval	118 (22)	**No**
			Informed Consent	135 (25)	**No**
			Ethics Approval and Informed Consent	74 (14)	**No**
138	Health Sciences	6 (434)	Conflicts of Interest Disclosure per Journal	98-100%	**Yes**
			Funding Disclosure per Journal	41-100%	**For some items**
44	Health Sciences	27 (304)	Abstract Structure conforming to ItAs	For 26 out of 27 journals almost all abstracts conformed to ItA instructions	**Yes**
52	Health Sciences	3 (190)	Abstract Structure conforming to ItAs	20% to 56% of articles conformed to ItA instructions	**No**
55	Health Sciences	18 (71)	Conflicts of Interest Disclosure	11 (15)	**No**
135	Health Sciences	3 (36)	CONSORT Item Adherence	Item adherence ranged from 22% to 100%	**For some items**
120	Health Sciences	1 (28)	CONSORT-NPT Total Score	Overall adherence ranged from 21% to 57%	**No**
		1 (28)	CONSORT-NPT item Adherence	Adherence ranged from 0% to 100%	**For some items**
		1 (24)	STRICTA Total Score	Overall adherence ranged from 41% to 100%	**No**
		1 (24)	STRICTA item Adherence	Adherence ranged from 13% to 96%	**For some items**
102	Health Sciences	6 (62)	CONSORT Flow Diagram	20 (32)	**No**
			Trial Registration	3 (5)	**No**
104	Health Sciences	51 (NR)	Trial Registration	20 (39%) of journals had articles that did not mention trial registration	**No**

Acronyms: CONSORT - The Consolidated Standards of Reporting Trials Statement, CONSORT-NPT – CONSORT extension for Non-Pharmacological Trials, RCTs – Randomized Controlled Trials, STRICTA - Standards for Reporting Interventions in Clinical Trials of Acupuncture.

Section 2. Series of Meta Analyses

For each topic we meta-analysed, we first present overall findings and possible differences between different disciplines, and then effects found only in studies that analysed *Health Sciences* journals, as most studies of Instruction to Authors (ItAs) were conducted on *Health Sciences* journals. In order to preserve the same reference order as in the paper, references[1-20] represent those used in the introduction and methods, while references [21-173] refer to the 153 studies that were included in our systematic review and meta-analyses. As time trends were estimated using regression models, related reported percentages may somewhat differ from the percentages reported in individual studies. All data is available at: 10.17632/53cskwwpdn.4.

Results Per Topic

Authorship

Twenty-six publications analysed if authorship was addressed in ItAs[28, 40, 54, 57, 75, 76, 81, 82, 88, 91, 97, 98, 109, 112, 119, 121, 123, 124, 134, 137, 143, 144, 147, 149, 165, 166]. These studies reported 121 authorship percentages (AP, defined as a number of journals whose ItAs addressed authorship divided by a total number of journals analysed in a study). Of these, almost half APs (n=51, 42%) were reported in one study which analysed journals published in 2010 and listed in *Science Citation Index* (SCI), *Social Science Citation Index* (SSCI), and *Arts & Humanities Citation Index* (A&HCI) categories. Specifically, the study analysed: a) top five journals assigned to several sub-disciplines of SCI and SSCI, and b) a random third of journals assigned to selected sub-disciplines of A&HCI.[98] Meta-analysis of these APs showed considerable heterogeneity between disciplines (I^2=94%; Q-value=84.6, df=5, p<0.001; Supplementary Figure 1), as well as a great variability between sub-disciplines (i.e. in *Physical Sciences* discipline all five top journals belonging to the category *Materials Science, multidisciplinary* addressed the topic of authorship, while none did so in *Statistics & Probability*, or

Telecommunications sub-disciplines). Overall, authorship was more often addressed in *Multidisciplinary Sciences*, *Health Sciences* and *Life Sciences* journals, than in *Social Sciences*, *Physical Sciences* or *Arts & Humanities* journals (subgroup analysis of APs by discipline). In addition, journals of multidisciplinary subdisciplines were significantly more likely to address authorship.

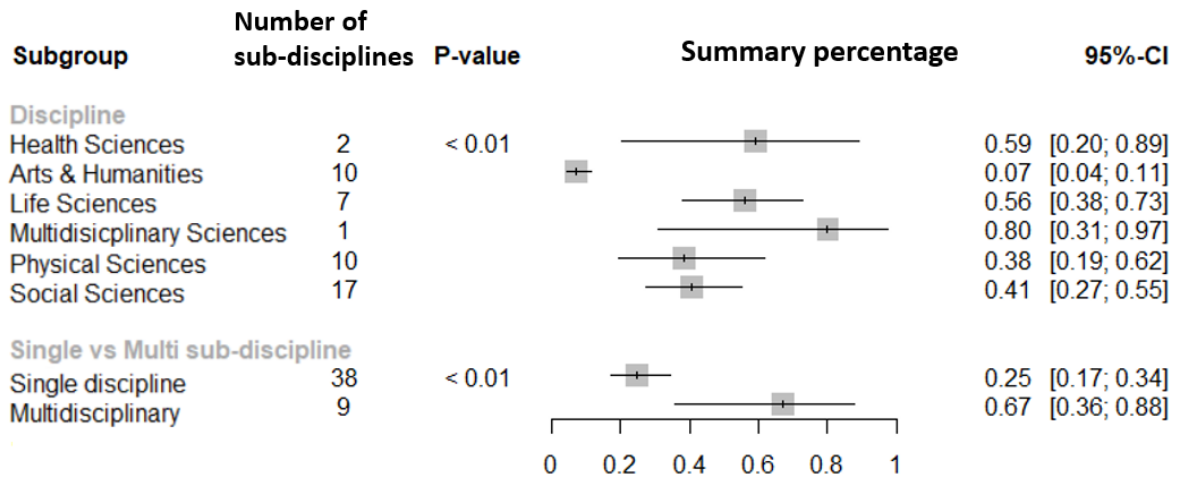

Supplementary Figure 1. Subgroup analyses for proportion of journals addressing authorship in their instructions to authors. Analyses are pooled by discipline, or single vs multidisciplinary nature of their subdiscipline category (subdisciplines were chosen from *Science Citation Index* (SCI), *Social Science Citation Index* (SSCI), and *Arts & Humanities Citation Index* (A&HCI) in 2010[98]). Shown are sub-disciplines' summary percentages with 95% CIs. Short vertical lines represent point estimate, horizontal lines represent 95% CIs and size of grey rectangle represent weight used in a meta-analysis model. Subgroups demonstrated large heterogeneity so no pooling of overall summary AP was performed.

Two studies by the same author compared different disciplines within a country, specifically of open access journals in Croatia, analysing almost the same set of journals, but applying different coding, data extraction methods, and journal classification.[143, 144] The first study compared *Health* and *non-Health* journals in 2014 and reported APs of 29% (95% CI 17-45%) vs 11% (95% CI 7-17%) demonstrating large heterogeneity of percentages (I^2=87%, Q-value=7.7, df=1, p=0.005),[143] while the second study compared *Health Sciences*, *Natural Sciences*, *Technical Sciences*, *Bio-Technical Sciences*, *Social Sciences*, and *Arts & Humanities* in 2015, and displayed less heterogeneity (I^2=64%, Q-value=14.1, df=5, p=0.015), with

authorship more frequently addressed in *Health Sciences* journals (AP of 37%, 95% CI 22-55%) than in *Arts & Humanities, Social Sciences*, or *Technical Sciences* (APs of *Health Sciences* were 8 to 29% higher, p≤0.045). In addition to discipline and country-specific APs, these two studies and one additional study,[134] also reported aggregate (i.e. cross-discipline) APs for Croatia, which showed steady, but not statistically significant increase in APs per year (9% in 2013, 14% in 2014, and 18% in 2015; p≥0.073 for difference between 2013 and 2014, and P=0.269 for difference between 2014 and 2015).

APs of *Social Sciences* journals were reported in 3 studies (Croatia in 2013,[124,] and 2015,[144] and Spain and Latin-America in 2015[149]) and demonstrated significant differences between countries (subgroup analysis, Q-value=11.9, df=2, p=0.001) with the Croatian summary AP estimate being significantly lower (10% vs 25%, AP difference of 15%, 95% CI 6-25%).

Authorship in Health Sciences journals

Twenty-one studies reported APs in *Health Sciences* journals [28, 40, 57, 75, 76, 81, 82, 88, 91, 97, 98, 112, 119, 121, 123, 137, 143, 144, 147, 165, 166]. However, they differed greatly in analysed databases and journal selection methods and were highly heterogeneous (I^2=84%, Q-value=25.0, df=4, p<0.001) and. Therefore, we explored the sources of heterogeneity based on available data, and found that time and (database) indexation affected the likelihood of addressing authorship in *Health Sciences* journals (Supplementary Figure 2). Namely, top journals or journals index in *Abridged Index Medicus* (AIM), between 1995 (AP 46%) and 2010 (AP 86%) demonstrated a 3% annual increase (14% annual odds increase, p for meta-regression coefficient, $P_{M\text{-}RC}$=0.035, and pseudo-R^2=68%; however pseudo-R^2 dropped to 0% when journals indexed in Index Medicus, IM, were included in the model). Additionally, one study reported on the difference

between WAME member journals (70%) and non-member journals (40%) in 2016 (AP difference, 30%, 95% CI 17-42%).[57]

Country/region specific APs were reported in 10 studies,[76, 82, 97, 112, 119, 143, 144, 147, 165, 166] and demonstrated statistically significant differences (subgroup analysis by countries, Q-value=112.6, df=7, p<0.001, Supplementary Figure 2). Majority of country/region specific APs were lower than 50%, with the exception of China which reported the highest AP (86%), India (summary AP of 65%, (95% CI 58-71%; heterogeneity I^2=2%, Q-value=2.0, df=2, p=0.362), and South East Europe region (64%). In Iran, statistically significant differences were reported for journals published in different languages (AP difference of 19% of English vs Farsi, 95% CI 8-30%).[112] (Note: The specific AP rates of *Health Sciences* journals are a good model to depict the sensitivity of APs to various factors. Alongside the above differences between countries and publication language of a journal, in two studies from Croatia[143, 144] and India[165, 166], where researchers assessed a largely overlapping set of journals just one year apart, we observed a non-significant increase in AP of 8% in Croatia, and 12% decrease in India. This indicates possible influences of journal selection and analytic methods.) Additionally, the study in China in 2014 reported no statistically significant differences between Chinese Medical Association Publishing House (CMPAH) Health Sciences journals and non-CMPAH journals (summary AP 85%, 95% CI 80-89%).[147]

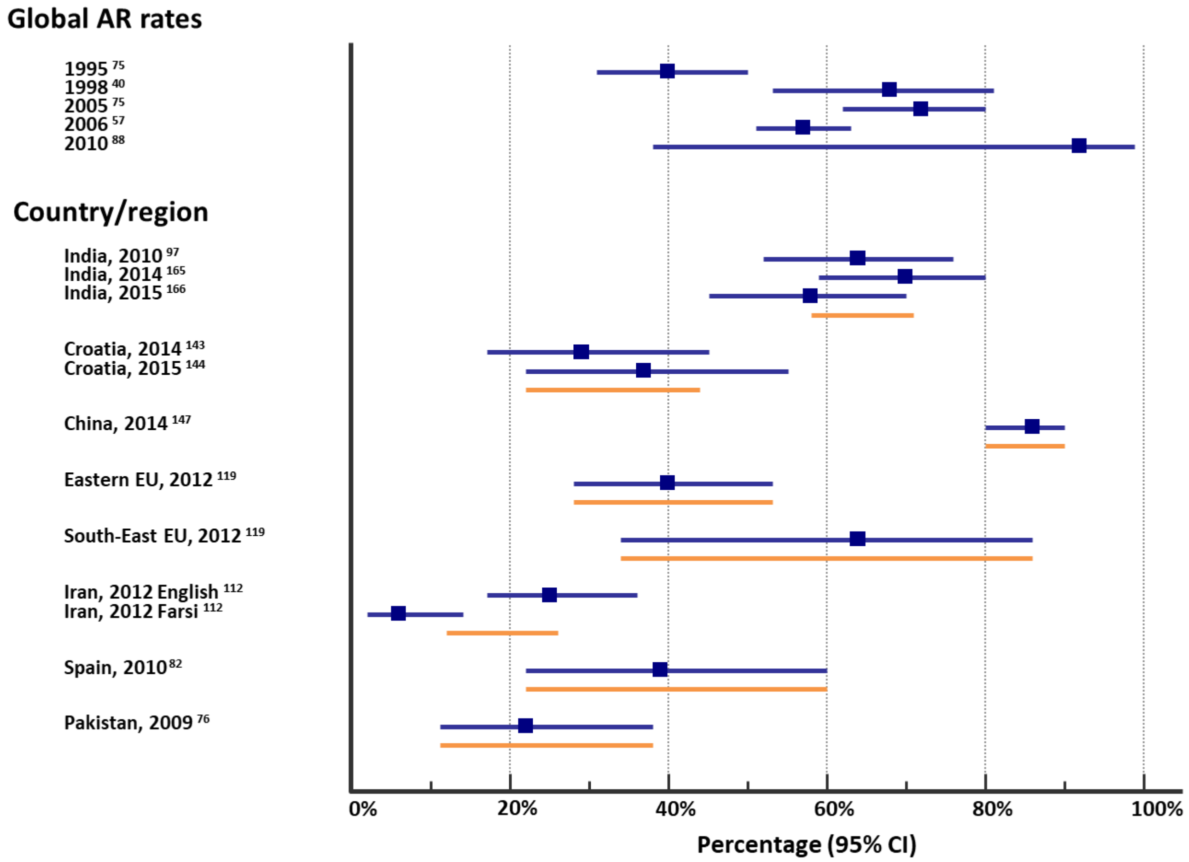

Supplementary Figure 2. Instructions to authors of *Health Sciences* journals addressing authorship based on journal indexation in international databases (global rates) or per country/region. Authorship reporting percentages (ARs) with 95% CI are shown in blue, while summary effects (per country) are shown in orange. Blue rectangles represent point estimates of percentages that were reported in primary studies, with size of a rectangle representing weight used in a meta-analysis model. Blue lines around rectangles represent 95% CIs of these point estimates. Orange lines correspond to 95% CI for country-specific summary effects. For global AR rates where meta-regression identified significant effect of time, the data were not pooled together. For similar reason, we did not pooled together data from different countries. References are shown as superscript numbers.

Finally, seven studies reported 12 APs of various *Health Sciences* subdisciplines (e.g. *Anaesthesiology*, *General Medicine*, *Plastic Surgery*, *Pharmacy*, etc.) [28, 81, 91, 98, 121, 123, 137] They demonstrated moderate heterogeneity (I^2=43%, Q-value=19.1, df=11, p=0.059) with the summary AP of 52% (95% CI 41-63%). No time trend was detected for a period of 1995 to 2015 (meta-regression, $P_{M\text{-}RC}$=0.219).

One of these studies, in 2013, showed no significant differences between MEDLINE indexed Health Sciences pharmacy journals and non-indexed journals (summary AP 52%, 95% CI 35-69%), nor between PMC indexed and non-indexed journals (summary AP 52%, 95% CI 35-68%), nor between ICMJE endorsing and non-endorsing journals (summary AP 51%, 95% CI 34-68%). (Note: As can be seen from the wide range of CIs, the study was slightly underpowered due to study groups consisting of 11 to 22 journals).[121]

Association between citation metrics and addressing of authorship in Health Sciences journals

Two studies reported APs for journals grouped according to their impact factor (IF). One analysed journals from plastic surgery, oral-craniomaxillofacial/facial plastic surgery, and otorhinolaryngology/head and neck surgery in *Journal Citation Reports* (JCR).[81] Reanalysis of this data showed that journals with IF values 1-2 exhibited significantly higher APs that those with IF<1 (AP difference of 35%, 95% CI 4-57%, p=0.024), but there was no statistically significant difference regarding the top IF>2 category (p≥0.276) possibly due to small number of journals (n=6) in that category. The other study analysed nine general medicine journals with only three journals per each category (<1, 1-2.2, >2.2).[91] It showed stable, time invariant differences, from 2000 to 2010 between journals with IF <1 and >2.2 (0 out of 3 journals for IF<1 and 3 out of 3 journals for IF>2.2 throughout the years, with no changes for IF 1-2.2 category (2 out of 3 journals in 2000, 2005 and 2010).[91] One additional study (which did not report data in a way that would allow for meta-analysis) reported no association between IF of the journal and the addressing of authorship.[165]

Conflicts of interest

Thirty-six publications analysed if conflicts of interest (COI) were addressed in ItAs.[22, 28, 31, 34, 36, 40, 54, 55, 70, 72, 75, 77, 80, 81, 88, 90, 91, 99, 103, 109, 112, 114, 115, 119, 121, 137-140, 143-145, 147, 149, 151, 165]

These studies reported 112 COI percentages (CPs, defined as a number of journals whose ItAs addressed conflicts of interest divided by a total number of journals analysed in a study). Only three studies, however, reported CPs across disciplines. The first one, in 1996, analysed top 1396 journals ranked by IF or the number of times journals were cited, and reported a CP of 16% (95% CI 14-18%).[34] The second, in 1998, analysed 41 journals (mostly *Multidisciplinary, Health, and Life Sciences* journals which had previously published retractions or corrections due to misconduct cases), and reported a CP of 41% (95% CI 28-57%).[40] Finally, the third study, analysed top five *Health and Life Sciences* journals in 2010, and reported a CP of 100% (95% CI 38-100%).[88] We observed a rise in CPs with time, with the increase of 26% (95% CI 12-41%) between 1997 and 1998, and of 59% (95% CI 13-72%) between 1998 and 2010 (Note: this observation may be influenced by the fact that the latter two studies had much smaller sample sizes than the first, 41 and 5, vs 1396 of the first study, and they had a higher prevalence of health journals, which we show below have much higher percentages of CPs).

Two studies by the same author of open access journals in Croatia compared differences between disciplines, but had slight differences in journal selection and text analysis.[143, 144] The first study in 2014 compared *Health* and *non-Health* journals and reported CPs of 32% (95% CI 19-48%) vs 4% (95% CI 2-8%) demonstrating large heterogeneity between the groups (I^2=95%, Q-value=20.6, df=1, $p<0.001$),[143] while the second study in 2015 compared *Biomedical, Natural, Technical, Bio-Technical, Social Sciences, and Humanities*, and also showed considerable heterogeneity (I^2=77%, Q-value=21.8, df=5, P=0.001) with *Humanities* having the lowest CP of 7%, 95% CI 4-15% ($p\leq0.009$ for all comparisons except with Bio-Technical Sciences).[144] The highest CP of 43% (95% CI 27-61%) was observed for *Biomedical Sciences* ($p\leq0.045$ for comparisons with *Technical, Social Sciences or Humanities*). The yearly increase in country's overall, cross-discipline CPs was considerable, from 9% (95% CI 6-14%) in 2014 to 21% (95% CI 17-26%) in 2015 (mean difference 12%, 95% CI 19-33%). (Note: the

difference in analytic methods and sampling between these two studies might had an effect on this significant increase in CPs).

One additional study reported a country's overall, cross-discipline CPs. That of 9 journals from Cameroon in 2009 with reported CP of 22% (95% CI 6-58%).[72] Subgroup analysis showed a non-significant difference between Cameroon and Croatia (Q-value=0.3, df=1, p=0.562). (Note: the lack of effect is likely due to the small number of Cameroonian journals analysed).

Finally, one study analysed Spanish and Latin American *Social Sciences* journals in 2015, and reported a CP of 25% (95% CI 18-34%).[149] Subgroup analysis also showed a non-significant difference of -1% when compared with *Croatian Social Sciences* journals in 2015 (CP of 24%, 95% CI 16-34%).

Conflicts of interest in Health Sciences Journals

Thirty-three publications reported CPs in *Health Sciences* journals. [22, 28, 31, 36, 40, 54, 55, 70, 75, 77, 80, 81, 88, 90, 91, 99, 103, 109, 112, 114, 115, 119, 121, 137-140, 143-145, 147, 151, 165] The CPs of core *Health Sciences* journals indexed in AIM, analysed in two studies at three time points,[22, 75] changed from 10% (95% CI 5-17%) in 1986, to 94% (95% CI 88-97%) in 2005 (Supplementary Figure 3; the time trend was confirmed by non-overlapping 95% CIs). Similarly, top *Health Sciences* journals, analysed in the period between 1986 (CP 21%) and 2010 (CP 83%) in five studies (3 studies of exclusively *Health Sciences* journals,[22, 36, 70] and 2 that included some *Life* or *Multidisciplinary* journals[40, 88]) also showed steady increase in CP with time (a 13% annual increase in odds of addressing COI; $p_{M\text{-}RC}$ on time of 0.002; pseudo-R^2 of 76%, Supplementary Figure 3). Moreover, CPs of top journals across disciplines in the period 1997-1998 (16% and 41%, respectively),[34, 40] were lower than those of *Health Sciences* journals published at the

closest time periods (1995 and 2001), which even then already reached CPs of 53% and 75%.[36, 75]

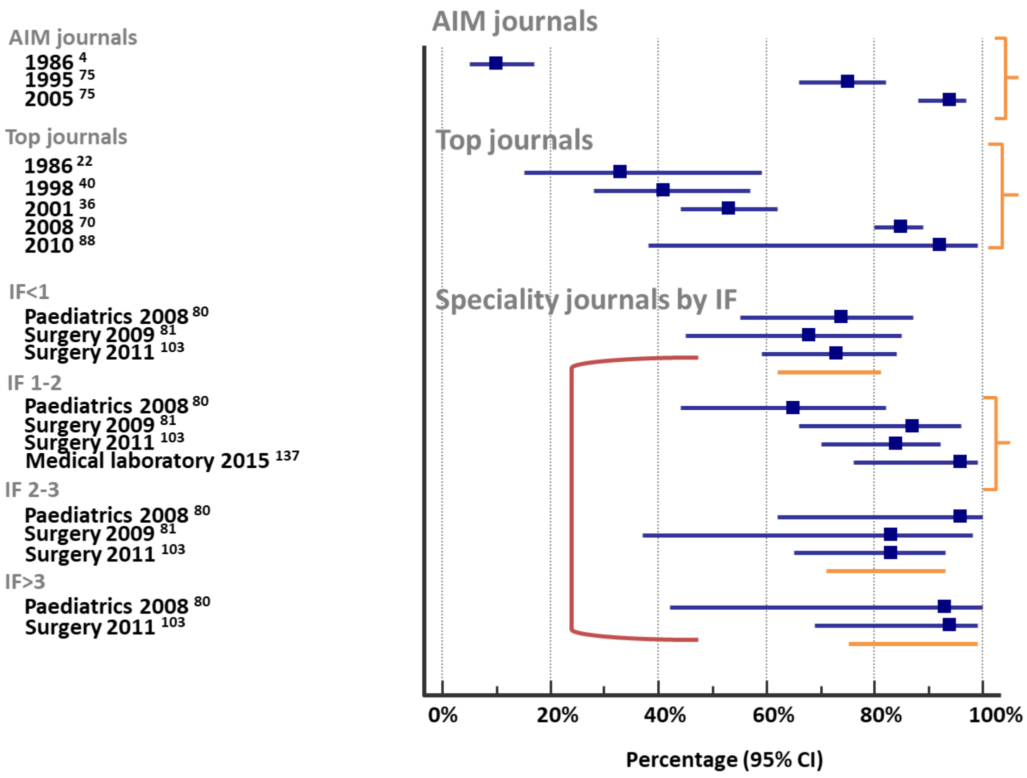

Supplementary Figure 3. Percentages of instructions to authors of *Health Sciences* journals addressing conflicts of interest. Journals are grouped into those listed in Abridged Index Medicus (AIM) database, classified as top Biomedical journals, or Specialty journals grouped by impact factor (IF) categories: IF<1, IF1-2, IF2-3, IF>3. Percentages with 95% CIs are shown in blue, with the blue rectangles representing point estimates that were reported in a study and size of rectangles representing weights used in a meta-analysis model. Summary effects including 95% CIs are shown in orange. For groups of studies where we found significant effect of time, marked with orange brackets, we did not pool the data. Significant difference in percentages between IF categories is marked with a red bracket. References are shown as superscript numbers.

Ten studies reported 49 CPs for *Health sub-disciplinary* journals, [28, 80, 81, 90, 91, 103, 121, 137-139] of which only 12 CPs belonging to 4 studies addressed the same subdiscipline two or more times (Supplementary Figure 3). Since the initial heterogeneity of CRs of *Health sub-disciplinary* journals was substantial (I^2=68%, Q-value=33.9, df=11, p<0.001) we performed a

subgroup analysis which demonstrated that pharmacy journals published in 2013 had significantly lower CPs (42%, 95% CI 27-60%) compared to summary CPs of clinical specialities between 1995 and 2015 (79%, 95% CI 75-85%) or general medicine between 2000 and 2010 (74%, 54%-87%) journals (subgroup analysis, Q-value=16.3, df=2, p<0.001). Still, the heterogeneity within the clinical speciality group was moderate to large (I^2=52%, Q-value=14.5, df=7, p=0.043). One study,[90] which sampled clinical speciality journals from the DOAJ database while most others sampled from the JCR sub-disciplines, increased this heterogeneity by ΔI^2=30% and its CP estimate of 63% (95% CI 48-77%) was significantly lower from that of the same sub-disciplines of journals sampled using JCR one year earlier (78%, 95% CI 67-86%; difference in rates of 15%, 95% CI -2% to 31%, significance at 0.1 level, p=0.091). We therefore, excluded it from pooling of the results. Summary CPs of clinical speciality journals from the remaining 7 studies demonstrated a CP increase with time, with the annual increase in odds of addressing COI of 9% (p_{M-RC} on time of 0.022, pseudo-R^2=100%) with CP from 64% in 1995 to that of a maximum of 96% (95% CI 76-99%) in 2015.

The study that was excluded, also reported significant differences for three groups of journal publishers (open access publishing houses, other, and professional organisation publishers, subgroup analysis Q=5.5, df=2, p=0.065, significant at 0.1 level).[90]

One of the included studies, in 2013, showed no statistically significant differences between MEDLINE indexed Health Sciences pharmacy journals and non-indexed journals (summary CP 43%, 95% CI 27-60%), nor between PMC indexed and non-indexed journals (summary CP 43%, 95% CI 27-60%), nor between ICMJE endorsing and non-endorsing journals (summary CP 43%, 95% CI 27-60%). (Note: As can be seen from the wide range of CIs, the study was slightly underpowered due to study groups consisting of 11 to 22 journals).[121]

Eleven country specific CPs for *Health Sciences* journals were reported in 10 studies,[31, 77, 112, 114, 115, 119, 143, 144, 147, 165] and showed large heterogeneity (I^2=94%, Q-value=154.6, df=10,

p<0.001). In India a significant increase in CPs was reported between 2008 and 2014 (from 30% to 89%, mean increase of 59%, 95% CI 44-70%). We also observed increases in CPs in Brazil between 2007 and 2012 (from 55% to 73%, CP increase of 18%, 95% CI from -6% to 41%), and in Croatia between 2014 and 2015 (from 32% to 43%, CP increase 12%, 95% CI -11% to 33%). (Note: absence of statistically significant differences in Croatia and Brazil could stem from high uncertainty of these estimates).

Additionally, national Iranian journals published in 2012 displayed significant differences between journals published in English (79%) and Farsi (31%) with CP difference of 48% in, 95% CI 33-60% respectively).[112] No difference was found in 1995 between ICMJE endorsing Mexican Health Sciences journals (CP 9%, 95% CI 3-24%) and non-endorsing journals (CP 4%, 95% CI 1-18%).[31] Finally, for China in 2014, there were significant differences between Chinese Medical Association Publishing House (CMPAH) Health Sciences journals (CP 34% 95% CI 24-46%) and non-CMPAH journals (CP 6% 95% CI 3-10%).[147]

(Note: as both the sub-discipline and country effects were demonstrated in the paragraphs above, 6 studies that reported country specific sub-discipline percentages were not included in pooled analyses[54, 55, 109, 140, 145, 151]).

Association of citation metrics and addressing of conflicts of interest in Health Sciences journals

Seven studies reported CPs for different IF subsets of *Health Sciences* journals.[80, 81, 91, 99, 103, 114, 137] The effect of IF across all *Health* subdisciplines CPs was, however, analysed only in one study which compared two datasets of top *Health* journals published in 2009: those with

IF≥10 and journals with IF<10 presenting with very high median IF of 4.7; and reported a 100% CP for both categories (summary CP of 98%, 95% CI 89-100%).[99]

In Brazil, an effect of the IF was evident in ItAs published in 2012, as journals with IF exhibited 38% higher CP (95% CI 12-58%) than those without IF (92% vs 54%, respectively, p=0.004).[114]

Five studies reported *Health sub-disciplinary* journals CPs (Supplementary Figure 3).[80, 81, 91, 103, 137] Of these, the CPs reported in one study were based on a sample of 3 journals per IF category so we excluded it from the integrated analysis.[91] This study, which analysed journals in the period from 2000 to 2010, demonstrated time invariant CPs in the middle (IF 1-2.2) and upper (IF >2.2) categories (3 out of 3 journals in highest, and 2 out of 3 in middle category addressing COI), whereas in the lowest category (IF <1) one journal addressed COI in 2000, and two in 2005 and 2010. Remaining studies analysed ItAs between 2008 and 2015 and grouped journals within approximately the same IF categories: IF<1, 1-2, 2-3, >3, and showed low heterogeneity (I^2=26%, Q-value=14.9, df=11, P=0.187). Nevertheless, meta-regression still revealed steady increase of CP with the increasing category of IF, with a significant increase observed only between the lowest (IF<1, CP of 72%) and the highest (IF>3, CP of 85%) category (increase of 5.9 times in odds of addressing COI; p_{M-RC} IF>3 vs IF<1=0.046; pseudo-R^2=74%).

One additional study (which did not report data in a way that allowed for meta-analysis) reported no association between IF of the journal and the addressing of conflicts of interest.[165]

Data Sharing

Ten publications analysed if data sharing was addressed in ItAs and reported 22 data sharing percentages (DPs, defined as a number of journals whose ItAs addressed data sharing

divided by a total number of journals analysed in a study).[24, 36, 40, 65, 83, 88, 117, 143, 144, 164] Five studies which covered a time span of 1992-2010 reported on DPs of predominantly top journals across disciplines.[24, 36, 40, 83, 88] While the heterogeneity of these percentages was considerable (I^2=97%, Q-value=138.8, df=4, p<0.001), time almost entirely explained the observed between-study variability of DPs. Namely, an annual increase in odds of addressing data sharing was 23% (p_{M-RC} <0.001, pseudo-R^2=100%; Supplementary Figures 4A and 4B) with the DP of 15% in 1992 to 88% in 2010.[88]

The time effect, however, was not observed for subdiscipline journals from different disciplines (molecular biology and biochemistry,[65] substance abuse,[117] biodiversity conservation[164]) which displayed substantial heterogeneity (I^2=57%, Q-value=4.7, df=2, p=0.096) with DPs ranging from 20 to 74% (Supplementary Figure 4A). Additionally, one of these studies, (which did not report data in a way that would allow for meta-analysis) also reported increased odds for the existence of a journal's data sharing policy for gene expression microarray data in higher impact factor journals, and open access journals, with a decrease in odds for Oncology journals.[65]

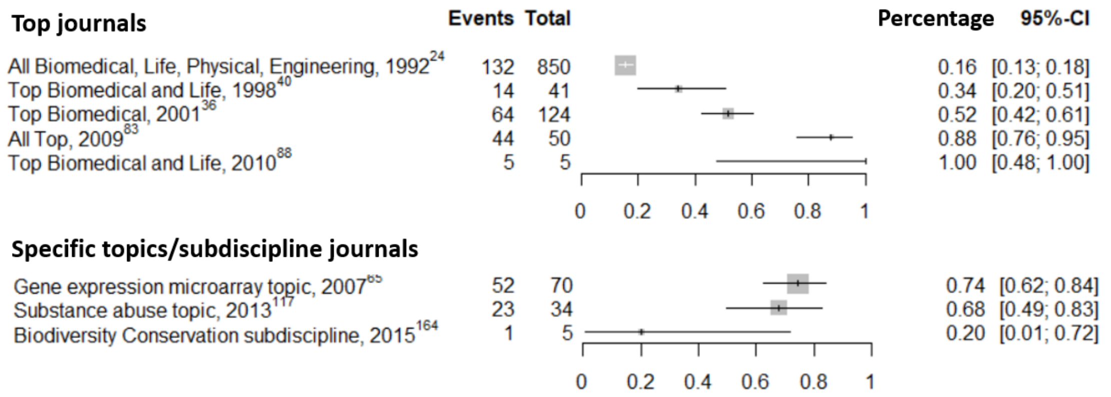

A

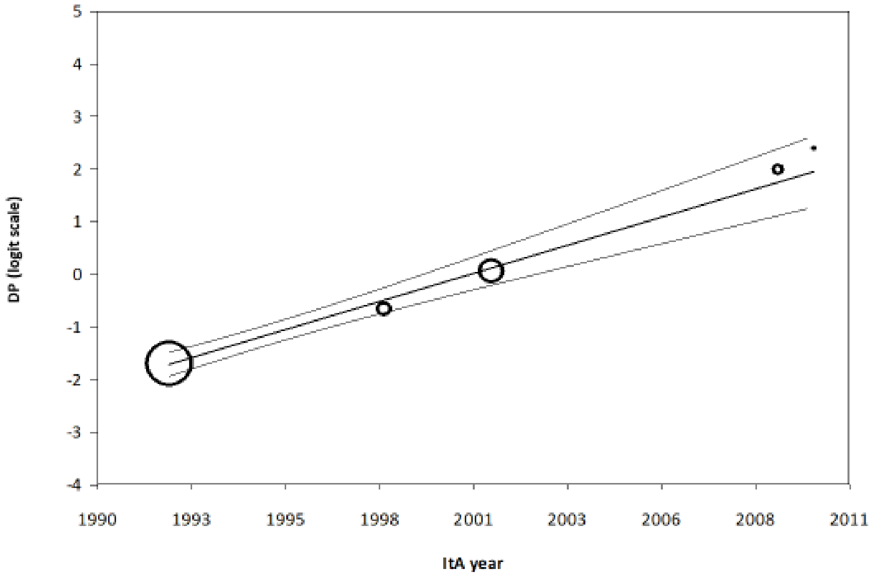

B

Supplementary Figure 4. Percentages of instructions to authors of journals addressing data sharing. A - Percentages of journals addressing data sharing in their instructions to authors, shown by category of journals and ordered by time. Percentages with 95% CIs are shown in blue, with the blue rectangle representing point estimate that was reported in a study and size of the rectangle representing weight used in a meta-analysis model. As we found significant effect of time and unexplained heterogeneity, we did not pool the data. References are shown as superscript numbers. B - Regression line illustrating how data sharing percentages (DP) have changed over time for top journals. Regression line is represented with full line, whereas 95% CI lines are shown in grey. Point estimates of the percentages that were reported in individual studies are shown as circles, with size of a circle representing weight used in a meta-regression model.

Two studies by the same authors compared differences between disciplines, specifically of open access journals in Croatia, analysing almost the same set of journals, but applying a different coding and extraction methods, and journal classification.[143, 144] The first study compared *Health* and *non-Health* journals in 2014 and reported DPs of 24% (95% CI 13-40%) vs 6% (95% CI 3-11%) demonstrating large heterogeneity of DPs (I^2=90%, Q-value=10.3, df=1, p<0.001),[144] while the *second* study compared *Biomedical*, *Natural*, *Technical*, *Bio-Technical*, *Social Sciences*, and *Humanities* in 2015, and also showed considerable heterogeneity (I^2=84%, Q-value=32.0, df=5, p<0.001),[143] with *Biotechnical* (DP of 77%) and *Natural Sciences* (DP of 62%) having the highest, and *Humanities* the lowest DPs (DP of 19%,

$p \leq 0.045$ for comparison with other disciplines). The yearly increase in overall country's DPs was considerable, from 9% in 2014 to 36% in 2015 (mean difference 27%, 95% CI 19-33%).

Association between citation metrics and addressing of data sharing in journals

Only one study investigating journals publishing papers on substance abuse reported DPs for different IF quartiles,[117] with one step increase in quartile category increasing the odds of addressing data sharing by 127%, corresponding to 22% percent increase in DP per quartile ($p_{M\text{-}RC}=0.035$, pseudo-$R^2=100\%$). The top quartile had significantly higher DP than the lowest one (89% vs 38%, mean difference 51%, 95% CI 6-77%). Two additional studies (which did not report data in a way that would allow for meta-analysis) indicated that a stronger data policy was associated with higher impact factor values.[65, 83]

Ethics approval

Thirty-one publications analysed if requiring ethics or institutional review board approval (IRB) was addressed in ItAs, and reported 123 ethics approval percentages (EPs, defined as a number of journals whose ItAs addressed ethics approval divided by a total number of journals analysed in a study).[26, 27, 30, 40, 45, 58, 60, 67, 69, 72, 74, 75, 77, 86, 89, 91-93, 97, 99, 108, 114, 115, 119, 137, 143, 145, 149, 151, 165, 166] Only three studies reported EPs not exclusively based on *Health Sciences* journals. Two studies reported country-specific EPs across disciplines: a study of Cameroonian journals in 2009 which showed an EP of 22% (95% CI 6-58%),[72] and a study of Croatian open access journals in 2014, which reported EP 8% (95% CI 5-13%).[143] No significant difference was found between the countries, likely due to high uncertainty of the Cameroon estimate. Finally, the study of Spanish and Latin-American journals indexed in JCR Social Sciences in 2014 reported a EP of 13% (95% CI of 8-21%).[149] The Croatian study additionally compared *non-Health Sciences* (EP of 5%, 95% CI 3-10%) with that of *Health Sciences* (21%, 95% CI 11-37%) and found *non-Health* EP to be significantly lower.

Ethics approval in Health Sciences Journals

Five studies reported 6 EPs of predominantly *Health Sciences* journals,[26, 40, 60, 75, 99] demonstrating very high heterogeneity (I^2=92%, Q-value=66.2, df=5, p<0.001), with EPs of top journals[40, 99] being lower than those indexed in AIM[26, 60, 75] published four to five years earlier and with both categories of journals showing increase in EP with time (Supplementary Figure 5). We, therefore, performed meta-regression using time and journal categories (AIM or top) as covariates and found that both significantly affected EPs ($p_{M\text{-}RC}$ for both ≤0.0002, pseudo-R^2=100%). Annual increase in odds of addressing EP was 16%; whereas AIMs indexation increased the odds by 164% relative to top journals.

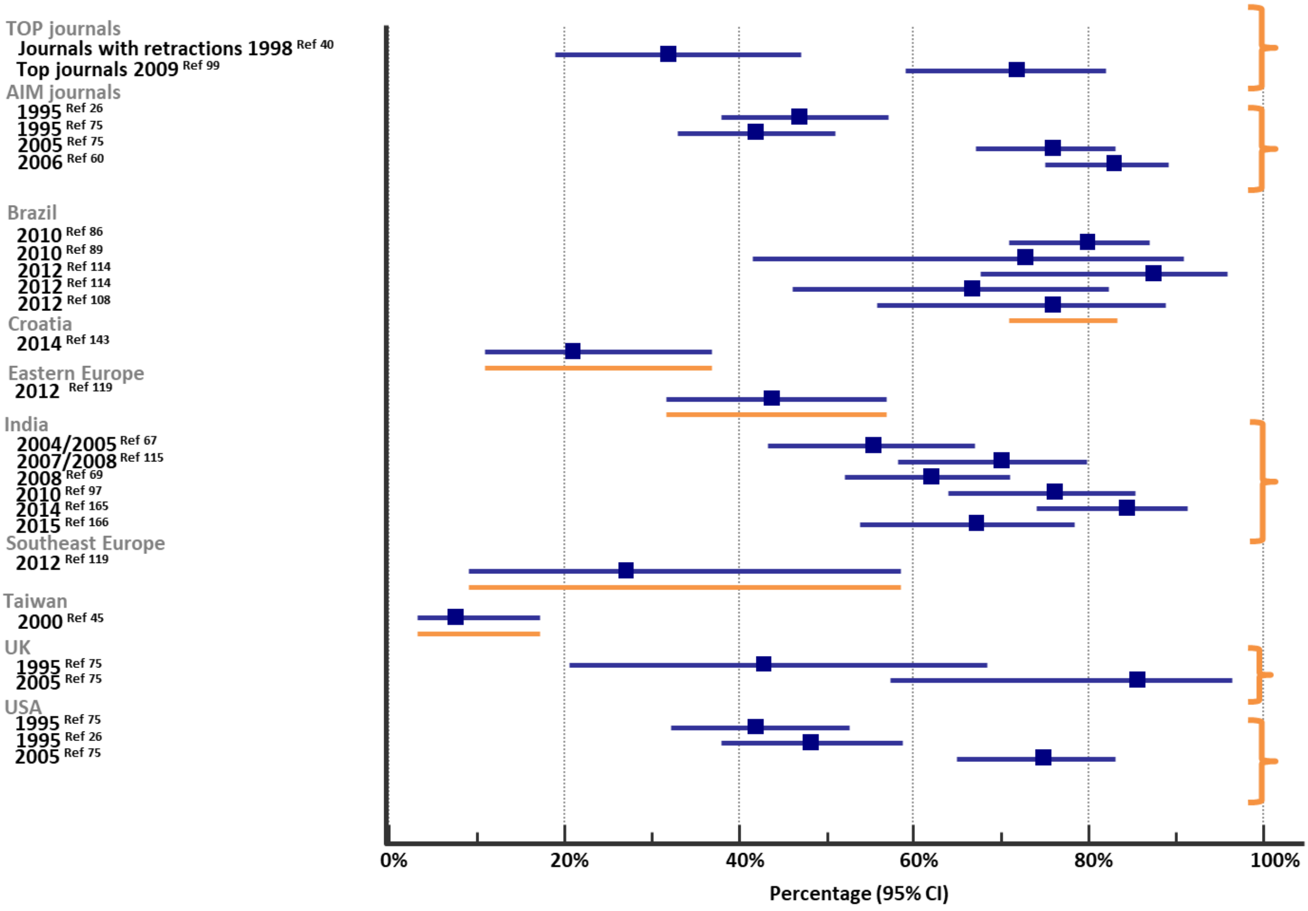

Supplementary Figure 5. Percentage of instructions to authors mentioning ethics approval in *Health Sciences* journals. Percentages with 95% CIs are shown in blue, with the blue rectangle representing point estimate that was reported in a study and size of the rectangle representing weight used in a meta-analysis model. Summary effects including 95% CIs are shown in orange. For groups of studies where we found significant effect of time, marked with orange brackets, we did not pool the data. References are shown as superscript numbers.

After excluding EPs calculated on sample sizes of ≤3 journals, seven studies remained which reported EPs in *Health Sciences* subdisciplines between 1995 and 2015.[26, 58, 74, 75, 91, 93, 137] These studies showed moderate heterogeneity (I^2=47%, Q-value=45.5, df=24, p=0.005), which was partly due to low precision of estimates, as more than half of reported EPs (13 out of 25, 52%) were derived from analyses based on ten or fewer journals. Nevertheless, we found that both time and subdiscipline affected EPs (meta-regression using time and sub-discipline as covariates, pseudo-R^2=100%). On average, the odds of addressing ethics approval in subdiscipline journals increased each year by 13% ($p_{M\text{-}RC}$ of time <0.001) which corresponds

to 3% annual percent increase. Further, compared to *Dentistry* journals as the reference category, *Internal Medicine* or *Surgery* increased the odds of addressing ethics approval in ItA by 9-12 times, which corresponded to 34-36% increase in EP (p_{M-RC} on speciality <0.001 for both). For AIM indexed subdisciplines: *Medicine*, *Miscellaneous*, *Paediatrics*, *Medical Laboratory Technology*, *Pathology,* and *Mixed* these odds were increased from 3.3 to 6.6 times ($p_{M-RC} \leq 0.079$); while *Nursing, Multidisciplinary* increased it by 2.1 times ($p_{M-RC}=0.031$). No statistically significant difference in odds compared to dentistry journals was found for *General Medicine,* and *Oral and Maxillofacial Surgery* subdisciplines.

Eighteen studies reported country or region specific EPs for *Health Sciences* journals,[26, 27, 45, 67, 69, 75, 77, 86, 89, 97, 108, 114, 115, 119, 143, 165, 166] and showed substantial heterogeneity between the countries/regions (subgroup analysis by countries, Q-value=119.7, df=8, p<0.001, Supplementary Figure 5). Nevertheless, in all the countries in which EPs were measured at two or more time points we observed an increase in EP with time. In India, the annual increase in odds of addressing ethics approval in ItAs between 2004/5 and 2015 was 5% (significant at 0.1 level, p_{M-RC} on time of 0.061, pseudo-R^2=47%), while in Brazil between 2007 and 2012, the increase in odds was 22% (significant at 0.1 level, p_{M-RC} on time of 0.074, pseudo-R^2=74%). In the UK, EPs increased by 43% (95% CI 7-67%, p=0.017) within a ten year period, while in the USA, in the same time period, EP increased by 27% (95% CI 12-40%, p<0.001) when comparing to the 1995 estimate reported by Amdur and Biddle,[26] or 33% (19-46%, p<0.001) when compared to a 1995 estimate reported by Rowan-Legg et al.[75] The study on Korean journals also reported percentages for subgroups of Medical Association member journals (EP 2%, 95% CI 0.3-10%) and quasi member journals (EP 5%, 95% CI 1-16%).[27]

One study on Indian journals in 2005 reported percentages for ICMJE endorsing Health Sciences journals (EP 79%, 95% CI 60-91%) and non-endorsing journals (EP 41%, 95% CI 28-57%), as well as Medline indexed Health Sciences journals and non-indexed journals (summary

EP 55%, 95% CI 43-67%).[67] Another study, by the same first author, looked at the almost the same journals in 2008 and reported percentages for ICMJE endorsing Health Sciences journals (EP 91%, 95% CI 71-97%) and non-endorsing journals (EP 61%, 95% CI 47-74%), as well as MEDLINE indexed Health Sciences journals and non-indexed journals (summary EP 70%, 95% CI 58-80%).[115]

(Note: as the sub-discipline, time and country effects were demonstrated in the paragraphs above, a study in India that reported on country specific sub-discipline percentages was not included in the pooled analyses.[151])

Association between citation metrics and addressing of ethics approval in journals

Two studies reported EPs in relation to IF values of journals.[91, 114] Of these, the EPs in one study were based on a sample of 3 journals per IF category so we excluded it from summary analysis.[91] This study, which analysed journals in the period from 2000 to 2010, demonstrated time invariant EPs for the upper (IF >2.2, 3 out of 3 journals requiring ethics approval), and middle category (IF 1-2, 0 out of 3 journals requiring ethics approval), whereas in the lowest category (IF <1) one journal required ethics approval COI in 2000 and 2005, and two journals in 2010. The second study, analysed the effect of IF on EPs in Brazil in 2012, and showed that EPs of JCR indexed journals with IF outperformed journals without IF that were sampled from Webqualis website by 21% (EPs of 88% vs 67%, 95% CI for difference from -3% to 42%, p=0.089 – significant at 0.1 level).[114] Four studies (which did not report data in a way that would allow for meta-analysis) reported on associations between IF and ethics approval, one demonstrating that higher impact factors journals were more likely to require ethics approvals,[75] while three others found no statistically significant associations with IF.[99, 137, 165]

Funding disclosure

Eleven publications analysed if funding or grants was addressed in ItAs, and reported 35 funding percentages (FPs, defined as a number of journals whose ItAs addressed funding disclosure divided by a total number of journals analysed in a study). [22, 23, 40, 54, 91, 115, 119, 138, 143, 149, 150] Only two studies reported FPs not exclusively based on *Health Sciences* journals: a study of Croatian open access journals in 2014, which for *non-Health Sciences* journals reported an FP of 12% (95% CI 8-18%) compared to *Health Sciences* of 37% (95% CI 23-53),[143] and that of Spanish or Latin American journals indexed in JCR Social Sciences in 2014, with an FP of 6% (95% CI 3-12%).[149] Both of these FPs were lower than any other country or sub-discipline specific FPs of *Health Sciences journals* (described below) which ranged from 28-93% ($p \leq 0.021$ for all comparisons).

Funding disclosure in Health Sciences journals

Two studies, reported five FPs for (predominantly) *Health Sciences* journals.[22, 40] One study, demonstrated in 1986 that FPs of non-indexed or randomly selected IM journals showed increased odds for addressing funding 5.43 times were significantly lower than those of AIM indexed or that of top journals. Subsequently, relative to non-indexed or IM journals, top or AIMs journals showed increased odds for addressing URM 18.88 times, corresponding to an increase in UP of 24% ($p_{M\text{-}RC}$ =0.001, pseudo-R2=94%, FPs of 3% and 1% vs 22% and 40% respectively, $p \leq 0.043$). Taken together, the two studies did not display significant increase in FPs of top journals between 1986 and 1998 (top journals summary estimate of 47%, 95% CI 34-60%; heterogeneity I^2=0%, Q-value=0.34, df=1, p=0.560), but did show that top journals were more likely to address funding in ItAs than AIM indexed journals which presented with

the estimated FP of 22%, 95% CI 15-31% (subgroup analysis by indexation, Q-value=10.1, df=1, p<0.001).

Three studies reported on country/region specific FPs,[115, 119, 143] with significant differences between them (subgroup analysis by region Q-value=23.1, df=3, p<0.001). The earliest reported FP of 70% (95% CI 58-80%), that of Indian journals in 2008, was also the highest (p≤0.028 for comparisons with other FPs), while those of Croatian open access journals in 2014, as well as those of Eastern and South-eastern European journals, in 2012, were comparable and ranged between 29-37%.

Three studies reported on FPs of *Health* sub-disciplinary journals [91, 138, 150] two of which estimated FPs on samples with fewer than ten journals, and were thus of low precision and heterogeneity (I^2=0, Q-value=2.0, df=4, p=0.732). Nevertheless, for the period between 2000 and 2015, they showed uniformity with the summary FP of 81% (95% CI 74-87%). (Note: as effects of country and subdisciplines were demonstrated above, one study,[54] which reported country specific sub-discipline FP was not included in the summary analyses.)

Association of citation metrics and disclosing of study funding in journals

Only one study, which analysed journals in the period from 2000 to 2010, demonstrated time invariant FPs for the upper (IF >2.2) and middle (IF 1-2) categories (3 out of 3 journals in the upper, and 2 out of 3 journals in the middle category requiring funding support disclosure), whereas in the lowest category (IF <1) 3 journals required funding support disclosure in 2000 and 2005, but only one in 2010.

Uniform Requirements for Manuscripts (URM)

Forty-five studies analysed if ItAs addressed any aspect of URM (known since 2013 as Recommendations for the Conduct, Reporting, Editing and Publication of Scholarly Work in Medical Journals, ICMJE Recommendations),[174] and reported a total of 141 URM percentages (UPs, defined as a number of journals whose ItAs addressed URM divided by a total number of journals analysed in a study).[22, 23, 26, 27, 30, 36, 41, 43, 45, 50, 54, 58, 60, 61, 67, 73-77, 80, 82, 85, 86, 90, 101-103, 108, 111, 115, 120, 121, 124, 126, 131, 134, 149, 150, 157, 162, 163, 165, 166, 173] Only four studies reported UPs of *non-Health Sciences* journals. Two of these analysed Croatian journals across all disciplines, and found country's UPs between 8% in 2008 (95% CI 3-8%) and 5% in 2013 (95% CI 5-13%).[85, 134] Two other studies, reported UPs for *Social Sciences* journals published within a particular region: Croatia in 2013 (2%, 95% CI 0-7%),[124] and Spain and Latin America in 2015 (15%, 95% CI 10-24%),[149] demonstrating high heterogeneity of percentages (I^2=89%, Q-value=9, df=1, P=0.003). Nevertheless, compared to UP of 61% for *Health Sciences* that were assessed on the comparable region and at comparable time - Latin America and Caribbean in 2012, *Social Sciences* UP from Spain and Latin America was significantly lower (difference of 45%, 95% CI 30-58%). Finally, one additional study, reported UPs of top 5 *Life* or *Health Sciences* journals from 4 different countries (Brazil, Mexico, Chile and Argentina) in 2013, and found no significant differences between them (UPs of 0-40%, subgroup analysis by country Q=2.1, df=3, p=0.558).[131]

Uniform requirements for manuscripts in Health Sciences journals

Six studies reported UPs for top *Health Sciences* journals indexed in JCR selected based on their IF values,[36, 41, 43, 50, 61, 162]. They showed that UPs significantly increased with time from 26% in 2001 to 76% in 2014 (meta-regression, $p_{M\text{-}RC}$ <0.001, pseudo-R2=83%, annual increase

in odds of addressing URM of 19%). One additional study reported UP for 15 top medical journals indexed in JCR in 1986, which was already very high (53%, 95% CI 29-76%), and was not included in the meta-regression analysis due to: a) much more stringent IF criteria applied to this set of journals compared to other estimates that were determined on least 8 times larger set of top journals, and b) the fact that its inclusion largely deteriorated the meta-regression model and reduced its pseudo-R2 by 35%.[22] This estimate was of very low precision, but was still significantly higher than the value for 124 top journals selected from the JCR list by IF in 2001,[36] or that of 100 randomly selected IM indexed journals assessed in 1986 ($p<0.007$ for both).[22]Additionally, one study, reported significant differences between CONSORT endorsing journals (UP 72%, 95% CI 56-84%) and non-endorsing journals (UP 35%, 95% CI 28-44%).[43]

Journals indexed in the AIM database, analysed in 5 studies,[22, 23, 26, 60, 75] showed a significant decrease from 35% in 1986 to 5% in 2006, with the annual decrease in odds for addressing URM of 9% ($p_{M\text{-}RC}<0.001$, pseudo-R^2=100%).

Differences between journals indexed in different databases were available only in one study[22] which reported UPs in 1986 being 6% (95% CI 1-34%) for non-indexed *Health Sciences* journals, 11% (95% CI 6-21%) for randomly selected IM indexed journals, 37% (95% CI 28-47) for AIM indexed journals, and 53% (95% CI 29-76%) for the top 15 *Health Sciences journals*. Subsequently, relative to non-indexed or IM journals, top or AIMs journals showed increased odds for addressing URM 5.43 times, corresponding to an increase in UP of 29% ($p_{M\text{-}RC}$ <0.001, pseudo-R2=100%).

One study in 2011 showed no statistically significant differences between *Health Sciences* journals that endorsed ICMJE or Consolidated Standards of Reporting Trials (CONSORT) standards (summary UP 71%, 95% CI 67-74%);[126] and it was comparable to that of top journals ranked by IF in 2014 (77%, 95% CI 70-83%).[162]

Seven studies reported UPs for *Health* sub-disciplinary journals listed in IM, JCR or DOAJ databases.[26, 80, 90, 101, 103, 150, 173]. The UPs reported between 2008 and 2016, irrespective of the indexing database or time, were quite homogeneous with the pooled estimate of 60% (95% CI 56-64%, heterogeneity I^2=0; Q-value=2.1, df=6, p=0.908). One study also reported significant differences for three groups of journal publishers in 2009 (open access publishing houses, other, and professional organisation publishers, summary UP of 65%, 95% CI 49-78%).[90] One additional study (which did not report data in a way that would allow for meta-analysis) also reported no association of medical association membership, publication language, and country of publication with Ups in 2010.[101]

Fourteen studies reported country or region-specific UPs. [26, 27, 30, 45, 67, 76, 77, 82, 86, 102, 111, 115, 165, 166] These studies showed great heterogeneity (Q=141.1, df=9, p<0.001). Given the effect of time observed with top *Health* journals and AIM listed journals, and the fact that time was also a significant modifier of UPs in India (p_{M-RC} for on time=0.074, pseudo-R2=66%; UPs from 4 studies demonstrated 7% annual increase in odds to address URM, from 54% in 2005 to 71% in 2015)[67, 115, 165, 166] the results from different countries were not summarized. The lowest UP was reported for Korea in 1997 (2%, 95% CI 0-6%),[27] while only two regions had UPs higher than 50% (Latin America and Caribbean in 2012 with UP 61%, 95% CI 47-73% ;[111] and India with 75%, 95% CI 63-83% in 2014,[165] and 67%, 95% CI 54-78% in 2015[166]). The study on Korean journals also reported percentages for subgroups of Medical Association member journals (UP 0%, 95% CI 0-7%) and quasi member journals (UP 2%, 95% CI 0.4-13%).[27] One study on Indian journals in 2005 reported percentages for ICMJE endorsing Health Sciences journals (UP 75%, 95% CI 55-88%) and non-endorsing journals (UP 49%, 95% CI 34-64%), as well as Medline indexed Health Sciences journals and non-indexed journals (summary UP 58%, 95% CI 46-70%).[67] Another study, by the same first author, looked at the almost the same journals in 2008 and reported percentages for ICMJE endorsing Health

Sciences journals (EP 91%, 95% CI 71-97%) and non-endorsing journals (EP 61%, 95% CI 47-74%), as well as MEDLINE indexed Health Sciences journals (EP 60%, 95% CI 45-72%) and non-indexed journals (EP 35%, 95% CI 18-57%).[115]

(Note: as specialty, database indexation, time and country effects were demonstrated in the paragraphs above, studies that reported combination of those characteristics were not included in pooled analyses. [54, 58, 73, 74, 108, 120, 121, 157, 163]) One of these studies, in 2013, showed a statistically significant difference (at P=0.1) between MEDLINE indexed Health Sciences pharmacy journals (UP 60%, 95% CI 36-80%) and non-indexed journals (UP 83%, 95% CI 61-94%), no significant differences between PMC indexed and non-indexed journals (summary UP 73%, 95% CI 55-85%), and significant differences (P=0.028) between ICMJE endorsing (UP 93%, 95% CI 69-99%) and non-endorsing journals (UP 58%, 95% CI 36-77%). (Note: As can be seen from the wide range of CIs, the study was slightly underpowered due to study groups consisting of 11 to 22 journals). [121]

Association between citation metrics and addressing of URM in Health Sciences journals

Five studies reported UPs for *Health Sciences* sub-disciplinary journals in relation to their IF values.[80, 101, 103, 163, 173] These UPs were quite homogeneous (I^2=14%, Q-value=14.0, df=12, p=0.302) with the summary UP of 59% (95% CI 53-65%). Nevertheless, due to previously shown effects of time, we additionally performed meta-regression using time and IF category as covariates. After we grouped subdiscipline UPs by the average IF factor of journals UPs were assigned to into categories IF<1, ≥1-2, ≥2-3, ≥3; we found a significant effect of IF on UPs of journals belonging to the highest, in comparison to the lowest IF category ($p_{RC\ IF\geq3\ vs\ IF<1}$ =0.033, pseudo-R2=49%). Namely, if a journal is assigned to the category IF≥3, its odds of addressing URM in ItAs increase by 2.8 times relative to the lowest category (UP of 74% vs 50%, respectively). Contrary, significant effect of time or lower IF categories on UPs were not

observed ($p_{M\text{-}RCs} \geq 0.261$). (Note: we cannot exclude that low heterogeneity of rates and non-significant effect of time might be attributed to underpowered analysis or a relatively short time span, between 2008 to 2016).

One study (which did not report data in a way that would allow for meta-analysis) reported no significant association between IF and UP.[165]

References:

1. Stevens A, Shamseer L, Weinstein E, Yazdi F, Turner L, Thielman J, et al. Relation of completeness of reporting of health research to journals' endorsement of reporting guidelines: systematic review. BMJ. 2014;348:g3804.
2. Leung V, Rousseau-Blass F, Beauchamp G, Pang DS. ARRIVE has not ARRIVEd: Support for the ARRIVE (Animal Research: Reporting of in vivo Experiments) guidelines does not improve the reporting quality of papers in animal welfare, analgesia or anesthesia. PLoS One. 2018;13(5):e0197882.
3. Chhapola V, Tiwari S, Brar R, Kanwal SK. Reporting quality of trial abstracts—improved yet suboptimal: A systematic review and meta-analysis. Journal of Evidence-Based Medicine. 2018;11(2):89-94.
4. Alluqmani A, Shamir L. Writing styles in different scientific disciplines: a data science approach. Scientometrics. 2018:1-15.
5. Argamon S, Dodick J, Chase P. Language use reflects scientific methodology: A corpus-based study of peer-reviewed journal articles. Scientometrics. 2008;75(2):203-38.
6. Marusic A, Bosnjak L, Jeroncic A. A systematic review of research on the meaning, ethics and practices of authorship across scholarly disciplines. PloS one. 2011;6(9):e23477.
7. Sollaci LB, Pereira MG. The introduction, methods, results, and discussion (IMRAD) structure: a fifty-year survey. J Med Libr Assoc. 2004;92(3):364-7.
8. Lin L, Evans S. Structural patterns in empirical research articles: A cross-disciplinary study. English for Specific Purposes. 2012;31(3):150-60.
9. Nambiar R, Tilak P, Cerejo C. Quality of author guidelines of journals in the biomedical and physical sciences. Learn Publ. 2014;27(3):201-6.
10. Bosch X, Hernández C, Pericas JM, Doti P, Marušić A. Misconduct policies in high-impact biomedical journals. PloS one. 2012;7(12):e51928.
11. Hauptman PJ, Armbrecht ES, Chibnall JT, Guild C, Timm JP, Rich MW. Errata in Medical Publications. Am J Med. 2014.
12. Malički M, Aalbersberg IJ, Bouter L, Ter Riet G. Journals' instructions to authors: A cross-sectional study across scientific disciplines. PloS one. 2019;14(9).
13. Ceprano MA, Stabile C. A comparative study of four reading journals' contributions to comprehension instruction methodology. Literacy Research and Instruction. 1986;25(2):108-15.
14. Olson CM, Jobe KA. Reporting institutional review board approval and patient consent. JAMA. 1997;278(6):477.
15. Wager E, Williams P, Project Overcome failure to Publish nEgative fiNdings C. "Hardly worth the effort"? Medical journals' policies and their editors' and publishers' views on trial registration and publication bias: quantitative and qualitative study. BMJ. 2013;347:f5248.
16. Curran WJ. The law and human experimentation. The New England journal of medicine. 1966;275(6):323-5.
17. Chalmers I, Glasziou P. Avoidable waste in the production and reporting of research evidence. The Lancet. 2009;374(9683):86-9.
18. Pupovac V, Fanelli D. Scientists Admitting to Plagiarism: A Meta-analysis of Surveys. Sci Eng Ethics. 2015;21(5):1331-52.
19. Katavic V. Retractions of scientific publications: responsibility and accountability. Biochem Med (Zagreb). 2014;24(2):217-22.
20. The STM Report: An overview of scientific and scholarly journal publishing. Hague, the Netherlands; 2018.
21. Else JF, Sanford MJ. Nonsexist language in social work journals: Not a trivial pursuit. Social Work. 1987;32(1):52-9.
22. Weller AC. Editorial policy and the assessment of quality among medical journals. Bulletin of the Medical Library Association. 1987;75(4):310.

23. Weller AC. The" Instructions to Authors" Section as an Aid in Serials Collection Development. The Serials Librarian. 1987;11(3-4):143-54.
24. McCain KW. Mandating sharing: Journal policies in the natural sciences. Science Communication. 1995;16(4):403-31.
25. Sosa-de-Martínez M, Carnevale A, Avendaño-Inestrillas J. Comparison of instructions to authors of Mexican medical journals and the Vancouver requirements. Revista de investigacion clinica; organo del Hospital de Enfermedades de la Nutricion. 1995;47(3):203-10.
26. Amdur RJ, Biddle C. Institutional review board approval and publication of human research results. JAMA. 1997;277(11):909-14.
27. Jeong I-S, Baik J-M, Jang I-J, Shin S-G. Ethical Guidelines Published in the Instruction for Authors of Biomedical Journals in Korea. Korean J Clin Pharmacol Ther. 1998;6(2):339-43.
28. Asai T, Shingu K. Ethical considerations in anaesthesia journals. Anaesthesia. 1999;54(2):192-7.
29. Marsh H, Eros CM. Ethics of field research: Do journals set the standard? Science and Engineering Ethics. 1999;5(3):375-82.
30. Sardenberg T, Müller S, Pereira H, Hossne W. Analysis of ethical aspects on human experimentation included in the instructions for the authors in 139 Brazilian scientific journals. Revista da Associacao Medica Brasileira (1992). 1999;45(4):295-302.
31. Sosa-de-Martínez M, Reyes-Miranda R, Pablos-Hach J, Avendaño-Inestrillas J, Martínez-Sosa M, Aragon-Gonzalez G. Norms for authors of Mexican medical journals in 1994 and 1995. Revista de investigacion clinica; organo del Hospital de Enfermedades de la Nutricion. 1999;51(4):235-44.
32. Tanahashi Y. Importance of author abstract and comparative analysis of journals' instructions to authors. Information Science and Technology Association. 1999;49(5):236-43.
33. Brown C. The E-volution of preprints in the scholarly communication of physicists and astronomers. Journal of the Association for Information Science and Technology. 2001;52(3):187-200.
34. Krimsky S, Rothenberg LS. Conflict of interest policies in science and medical journals: editorial practices and author disclosures. Science and engineering ethics. 2001;7(2):205-18.
35. Ferguson JA, Mockbee C, Erbele S, Muniz E. Evaluation of published case reports' standards and notification. Drug information journal. 2002;36(2):303-7.
36. Atlas MC. Emerging ethical issues in instructions to authors of high-impact biomedical journals. Journal of the Medical Library Association. 2003;91(4):442.
37. Bayne SC, McGivney GP, Mazer SC. Scientific composition and review of manuscripts for publication in peer-reviewed dental journals. Journal of Prosthetic Dentistry. 2003;89(2):201-18.
38. Brown C. The changing face of scientific discourse: Analysis of genomic and proteomic database usage and acceptance. Journal of the American Society for Information Science and Technology. 2003;54(10):926-38.
39. Casserly MF, Bird JE. Web citation availability: analysis and implications for scholarship. College & Research Libraries. 2003;64(4):300-17.
40. Scheetz M, editor Promoting Integrity Through "Instructions to Authors: A Preliminary Analysis. Proceedings of the 1st ORI Research Conference on Research Integrity Available at: http://6959; 2003.
41. Atlas MC. Retraction policies of high-impact biomedical journals. Journal of the Medical Library Association. 2004;92(2):242.
42. Sorinola O, Olufowobi O, Coomarasamy A, Khan KS. Instructions to authors for case reporting are limited: a review of a core journal list. BMC medical education. 2004;4(1):4.
43. Altman DG. Endorsement of the CONSORT statement by high impact medical journals: survey of instructions for authors. BMJ. 2005;330(7499):1056-7.
44. Nakayama T, Hirai N, Yamazaki S, Naito M. Adoption of structured abstracts by general medical journals and format for a structured abstract. Journal of the Medical Library Association. 2005;93(2):237.

45. Shih Y-T, Shih S-F, Chen N-S, Chen C-S. Human research protections-Current status in Taiwan and policy proposals. Taiwan Journal of Public Health. 2005;24(4):360-73.
46. Berhidi A, Geges J, Vasas L. The biomedical periodicals of Hungarian editions--historical overview. Orvosi hetilap. 2006;147(10):457-67.
47. Outram SM, Ellison GT. Improving the use of race and ethnicity in genetic research: a survey of instructions to authors in genetics journals. Sci Ed. 2006;29:78-81.
48. Poolman RW, Struijs PA, Krips R, Sierevelt IN, Lutz KH, Bhandari M. Does a" Level I Evidence" rating imply high quality of reporting in orthopaedic randomised controlled trials? BMC medical research methodology. 2006;6(1):44.
49. Puhan MA, Ter Riet G, Eichler K, Steurer J, Bachmann LM. More medical journals should inform their contributors about three key principles of graph construction. Journal of clinical epidemiology. 2006;59(10):1017-22.
50. Schriger DL, Arora S, Altman DG. The content of medical journal Instructions for authors. Annals of emergency medicine. 2006;48(6):743-9.
51. Woolley KL, Ely JA, Woolley MJ, Findlay L, Lynch FA, Choi Y, et al. Declaration of medical writing assistance in international peer-reviewed publications. JAMA. 2006;296(8):929-34.
52. Hartley J. Clarifying the sub-headings of structured abstracts. European Science Editing. 2007;33(2):41-2.
53. Ioannidis JP. Limitations are not properly acknowledged in the scientific literature. Journal of clinical epidemiology. 2007;60(4):324-9.
54. Pellizzon RdF, Montero EFdS, Población DA, Monteiro R, Castro RCF. Brazilian scientific journals in surgery. III: analysis of the instructions for authors based on Vancouver uniform requirements. Acta cirurgica brasileira. 2007;22(6):503-10.
55. Schneider N, Lingner H, Schwartz FW. Disclosing conflicts of interest in German publications concerning health services research. BMC health services research. 2007;7(1):78.
56. Smidt N, Overbeke J, de Vet H, Bossuyt P. Endorsement of the STARD Statement by biomedical journals: survey of instructions for authors. Clinical chemistry. 2007;53(11):1983-5.
57. Wager E. Do medical journals provide clear and consistent guidelines on authorship? Medscape General Medicine. 2007;9(3):16.
58. Axelin A, Salanterä S. Ethics in neonatal pain research. Nursing Ethics. 2008;15(4):492-9.
59. Casserly MF, Bird JE. Web Citation Availability. Library Resources & Technical Services. 2008;52(1):42-53.
60. Freeman SR, Lundahl K, Schilling LM, Jensen JD, Dellavalle RP. Human research review committee requirements in medical journals. Clin Invest Med. 2008;31(1):E49-54.
61. Hopewell S, Altman DG, Moher D, Schulz KF. Endorsement of the CONSORT Statement by high impact factor medical journals: a survey of journal editors and journal 'Instructions to Authors'. Trials. 2008;9(1):20.
62. Janosky JE. Statistical testing alone and estimation plus testing: Reporting study outcomes in biomedical journals. Statistics & Probability Letters. 2008;78(15):2327-31.
63. Mrkobrada M, Thiessen-Philbrook H, Haynes RB, Iansavichus AV, Rehman F, Garg AX. Need for quality improvement in renal systematic reviews. Clinical Journal of the American Society of Nephrology. 2008;3(4):1102-14.
64. Perry AE, Johnson M. Applying the Consolidated Standards of Reporting Trials (CONSORT) to studies of mental health provision for juvenile offenders: a research note. Journal of Experimental Criminology. 2008;4(2):165-85.
65. Piwowar HA, Chapman WW, editors. A review of journal policies for sharing research data. ELPUB; 2008.
66. Salvagno GL, Lippi G, Montagnana M, Guidi GC. Standards of practice and uniformity in references style. Clinical chemistry and laboratory medicine. 2008;46(4):437-8.
67. Tharyan P, Premkumar TS, Mathew V, Barnabas JP. Editorial policy and the reporting of randomized controlled trials: a survey of instructions for authors and assessment of trial reports in Indian medical journals (2004-05). National medical journal of india. 2008;21(2):62-8.

68. Xu L, Li J, Zhang M, Ai C, Wang L. Chinese authors do need CONSORT: reporting quality assessment for five leading Chinese medical journals. Contemporary clinical trials. 2008;29(5):727-31.
69. Bavdekar SB, Gogtay NJ, Chavan R. Reporting ethical processes: survey of 'instructions to authors' provided by Indian journals. Indian J Med Sci. 2009;63(6):260-2.
70. Blum JA, Freeman K, Dart RC, Cooper RJ. Requirements and definitions in conflict of interest policies of medical journals. Jama. 2009;302(20):2230-4.
71. Monteiro R, Brandau R, Gomes WJ, Braile DM. Trends in animal experimentation. Brazilian Journal of Cardiovascular Surgery. 2009;24(4):506-13.
72. Nchangwi SM, Asahngwa C, Chi PC. Ethical Considerations In Instructions To Authors Of Some Journals Published In Cameroon. Ebonyi Medical Journal. 2010;8(2).
73. Pengel LH, Barcena L, Morris PJ. The quality of reporting of randomized controlled trials in solid organ transplantation. Transplant international. 2009;22(4):377-84.
74. Pitak-Arnnop P, Sader R, Hervé C, Dhanuthai K, Bertrand J-C, Hemprich A. Reporting of ethical protection in recent oral and maxillofacial surgery research involving human subjects. International journal of oral and maxillofacial surgery. 2009;38(7):707-12.
75. Rowan-Legg A, Weijer C, Gao J, Fernandez C. A comparison of journal instructions regarding institutional review board approval and conflict-of-interest disclosure between 1995 and 2005. Journal of medical ethics. 2009;35(1):74-8.
76. Samad A, Khanzada TW, Siddiqui AA. Do the instructions to authors of Pakistani medical journals convey adequate guidance for authorship criteria. Pak J Med Sci. 2009;25(6):879-82.
77. Tavares-Neto J, Azevêdo ES. Ethics relevance in Brazilian medical journals. Revista da Associação Médica Brasileira. 2009;55(4):400-4.
78. Akhabue E, Lautenbach E. “Equal” contributions and credit: an emerging trend in the characterization of authorship. Annals of epidemiology. 2010;20(11):868-71.
79. Hooijmans CR, Leenaars M, Ritskes-Hoitinga M. A gold standard publication checklist to improve the quality of animal studies, to fully integrate the Three Rs, and to make systematic reviews more feasible. Altern Lab Anim. 2010;38(2):167-82.
80. Meerpohl JJ, Wolff RF, Niemeyer CM, Antes G, von Elm E. Editorial policies of pediatric journals: survey of instructions for authors. Archives of pediatrics & adolescent medicine. 2010;164(3):268-72.
81. Pitak-Arnnop P, Bauer U, Dhanuthai K, Brückner M, Herve C, Meningaud J-P, et al. Ethical issues in instructions to authors of journals in oral-craniomaxillofacial/facial plastic surgery and related specialities. Journal of cranio-maxillo-facial surgery. 2010;38(8):554-9.
82. Ruiz-Pérez R, Marcos-Cartagena D, Delgado López-Cózar E. Fulfilment of the criteria about scientific authorship in Spanish biomedical and health science journals included in Journal Citation Reports. Revista espanola de salud publica. 2010;84(6):809-25.
83. Alsheikh-Ali AA, Qureshi W, Al-Mallah MH, Ioannidis JP. Public availability of published research data in high-impact journals. PloS one. 2011;6(9):e24357.
84. Bennett C, Khangura S, Brehaut JC, Graham ID, Moher D, Potter BK, et al. Reporting guidelines for survey research: an analysis of published guidance and reporting practices. PLoS medicine. 2011;8(8):e1001069.
85. Bošnjak L, Puljak L, Vukojević K, Marušić A. Analysis of a number and type of publications that editors publish in their own journals: case study of scholarly journals in Croatia. Scientometrics. 2011;86(1):227-33.
86. Fernandes MR, Queiroz MCCAM, Moraes MRd, Barbosa MA, Sousa ALL. Ethical standards adopted by Brazilian journals of medical specialties. Revista da Associação Médica Brasileira. 2011;57(3):267-71.
87. Fleischhacker S, Evenson KR, Singh P, Rodriguez DA, Ammerman A. Does this study inform policy: examination of leading childhood obesity journals’ instructions to authors regarding policy-related research and implications. Childhood Obesity: Risk Factors, Health Effects and Prevention Hauppauge, New York: Nova Science Publishers. 2011:135-52.

88. Macrina FL. Teaching authorship and publication practices in the biomedical and life sciences. Science and engineering ethics. 2011;17(2):341-54.
89. Malafaia G, Rodrigues ASdL, Talvani A. Ethics in the publication of studies on human visceral leishmaniasis in Brazilian periodicals. Revista de saude publica. 2011;45(1):166-72.
90. Meerpohl JJ, Wolff RF, Antes G, von Elm E. Are pediatric Open Access journals promoting good publication practice? An analysis of author instructions. BMC pediatrics. 2011;11(1):27.
91. Miesle LM, Oblak TA, Shrode LD, Horton AM, editors. Analyzing the landscape of author Instructions for general medicine Journals: Past and Present. Current medical research and opinion; 2011: Informa Helathcare.
92. Moro JV, Rodrigues JSM, Andre SCS. Research involving human beings in instructions to authors in domestic scientific nursing magazines. Rev bioét (Impr). 2011;19(2):543-52.
93. Navaneetha C. Editorial policy in reporting ethical processes: A survey of 'instructions for authors' in International Indexed Dental Journals. Contemporary clinical dentistry. 2011;2(2):84.
94. Rands SA. Inclusion of policies on ethical standards in animal experiments in biomedical science journals. Journal of the American Association for Laboratory Animal Science. 2011;50(6):901-3.
95. Tao K-m, Li X-q, Zhou Q-h, Moher D, Ling C-q, Yu W-f. From QUOROM to PRISMA: a survey of high-impact medical journals' instructions to authors and a review of systematic reviews in anesthesia literature. PLoS One. 2011;6(11):e27611.
96. Tao T, Bo L, Wang F, Li J, Deng X. Equal contributions and credit given to authors in anesthesiology journals during a 10-year period. Scientometrics. 2011;91(3):1005-10.
97. Yadav P, Chavda N. Survey of "instructions to authors" of Indian medical journals for reporting of ethics and authorship criteria. 2011;8(1):36.
98. Bošnjak L, Marušić A. Prescribed practices of authorship: review of codes of ethics from professional bodies and journal guidelines across disciplines. Scientometrics. 2012;93(3):751-63.
99. Charlier P, Bridoux V, Watier L, Ménétrier M, de la Grandmaison GL, Hervé C. Ethics requirements and impact factor. Journal of medical ethics. 2012;38(4):253-5.
100. Hopewell S, Ravaud P, Baron G, Boutron I. Effect of editors' implementation of CONSORT guidelines on the reporting of abstracts in high impact medical journals: interrupted time series analysis. BMJ. 2012;344:e4178.
101. Kunath F, Grobe HR, Rücker G, Engehausen D, Antes G, Wullich B, et al. Do journals publishing in the field of urology endorse reporting guidelines? A survey of author instructions. Urologia internationalis. 2012;88(1):54-9.
102. Li X-q, Tao K-m, Zhou Q-h, Moher D, Chen H-y, Wang F-z, et al. Endorsement of the CONSORT statement by high-impact medical journals in China: a survey of instructions for authors and published papers. PLoS One. 2012;7(2):e30683.
103. Shantikumar S, Wigley J, Hameed W, Handa A. A survey of instructions to authors in surgical journals on reporting by CONSORT and PRISMA. The Annals of The Royal College of Surgeons of England. 2012;94(7):468-71.
104. Tulvatana W, Thinkhamrop B, Kulvichit K, Tatsanavivat P. Endorsement and implementation of high impact factor medical journals on the International Committee of Medical Journal Editors (ICMJE) policy of mandatory clinical trial registration. Asian Biomedicine. 2012;6(3):423-7.
105. Wang F, Tang L, Bo L, Li J, Deng X. Equal contributions and credit given to authors in critical care medicine journals during a 10-yr period. Critical care medicine. 2012;40(3):967-9.
106. Borrego Á, Garcia F, editors. Provision of supplementary materials in library and information science scholarly journals. Aslib Proceedings: New Information Perspectives; 2013: Emerald Group Publishing Limited.
107. Grant SP, Mayo-Wilson E, Melendez-Torres G, Montgomery P. Reporting quality of social and psychological intervention trials: a systematic review of reporting guidelines and trial publications. PloS one. 2013;8(5):e65442.

108. Malafaia G, Guilhem D, Talvani A. Do Brazilian scientific journals promote the adherence of Chagas disease researchers to internacional ethical principals? Revista do Instituto de Medicina Tropical de São Paulo. 2013;55(3):159-65.
109. Mathur V, Dhillon J, Kalra G, Sharma A, Mathur R. Survey of instructions to authors in Indian and British Dental Journals with respect to ethical guidelines. Journal of Indian Society of Pedodontics and Preventive Dentistry. 2013;31(2):107.
110. Panic N, Leoncini E, De Belvis G, Ricciardi W, Boccia S. Evaluation of the endorsement of the preferred reporting items for systematic reviews and meta-analysis (PRISMA) statement on the quality of published systematic review and meta-analyses. PloS one. 2013;8(12):e83138.
111. Reveiz L, Villanueva E, Iko C, Simera I. Compliance with clinical trial registration and reporting guidelines by Latin American and Caribbean journals. Cadernos de saude publica. 2013;29(6):1095-100.
112. Salamat F, Sobhani A-R, Mallaei M. Quality of publication ethics in the instructions to the authors of Iranian journals of medical sciences. Iranian journal of medical sciences. 2013;38(1):57.
113. Santos SMd, Noronha DP. Brazilian scientific journals in Social Sciences and Humanities indexed by SciELO database: formal aspects. Perspectivas em Ciência da Informação. 2013;18(2):2-16.
114. Teixeira RKC, Yamaki VN, Gonçalves TB, Botelho NM, Silva JACd. Does impact factor influence the ethics of the instructions provided to journal authors? Revista da Associação Médica Brasileira. 2013;59(3):280-4.
115. Tharyan P, George AT, Kirubakaran R, Barnabas JP. Reporting of methods was better in the Clinical Trials Registry-India than in Indian journal publications. Journal of clinical epidemiology. 2013;66(1):10-22.
116. Yoshida A, Dowa Y, Murakami H, Kosugi S. Obtaining subjects’ consent to publish identifying personal information: current practices and identifying potential issues. BMC medical ethics. 2013;14(1):47.
117. Aleixandre-Benavent R, Vidal-Infer A, Arroyo AA, Zurián JCV, Cañigral FB, Sapena AF. Public availability of published research data in substance abuse journals. International Journal of Drug Policy. 2014;25(6):1143-6.
118. Betini M, Volpato ES, Anastácio GD, Faria RT, El Dib R. Choosing the right journal for your systematic review. Journal of evaluation in clinical practice. 2014;20(6):834-6.
119. Broga M, Mijaljica G, Waligora M, Keis A, Marusic A. Publication ethics in biomedical journals from countries in Central and Eastern Europe. Science and engineering ethics. 2014;20(1):99-109.
120. Choi J, Jun JH, Kang BK, Kim KH, Lee MS. Endorsement for improving the quality of reports on randomized controlled trials of traditional medicine journals in Korea: a systematic review. Trials. 2014;15(1):429.
121. Courbon È, Tanguay C, Lebel D, Bussières J-F. Paternité des articles et intérêts concurrents: une analyse des recommandations aux auteurs des journaux traitant de pratique pharmaceutique. The Canadian journal of hospital pharmacy. 2014;67(3):188.
122. Fuller T, Peters J, Pearson M, Anderson R. Impact of the transparent reporting of evaluations with nonrandomized designs reporting guideline: ten years on. American journal of public health. 2014;104(11):e110-7.
123. Gasparyan AY, Ayvazyan L, Gorin SV, Kitas GD. Upgrading instructions for authors of scholarly journals. Croatian medical journal. 2014;55(3):271.
124. Grgić IH, editor IL and information ethics: how to avoid plagiarism in scientific papers? European Conference on Information Literacy; 2014: Springer.
125. Hoffmann T, English T, Glasziou P. Reporting of interventions in randomised trials: an audit of journal instructions to authors. Trials. 2014;15(1):20.
126. Hooft L, Korevaar D, Molenaar N, Bossuyt P, Scholten R. Endorsement of ICMJE’s Clinical Trial Registration Policy: a survey among journal editors. Neth J Med. 2014;72(7):349-55.
127. Nguyen JT, Shahid R, Manera R. Pediatric case reports: assessing recommendations from journals’ instructions to authors. Hospital pediatrics. 2014;4(1):39-43.

128. Sankar P, Cho MK, Monahan K, Nowak K. Reporting Race and Ethnicity in Genetics Research: Do Journal Recommendations or Resources Matter? Science and engineering ethics. 2014;21(5):1353-66.
129. Splendiani B, Ribera M. Accessible images in computer science journals. Procedia Computer Science. 2014;27:9-18.
130. Splendiani B, Ribera M, Garcia R, Termens M. Do physicians make their articles readable for their blind or low-vision patients? An analysis of current image processing practices in biomedical journals from the point of view of accessibility. Journal of digital imaging. 2014;27(4):419-42.
131. Valles EG, Bernacchi AS. Do Latin American scientific journals follow dual-use review policies? Biosecurity and bioterrorism: biodefense strategy, practice, and science. 2014;12(2):94-105.
132. Xiao L, Hu J, Zhang L, Shang H-c. Endorsement of CONSORT by Chinese medical journals: A survey of “instruction to authors”. Chinese journal of integrative medicine. 2014;20(7):510-5.
133. Aleixandre-Benavent R, Vidal-Infer A, Alonso-Arroyo A, de Dios González J, Ferrer-Sapena A, Peset F, editors. Open availability of articles and raw research data in spanish pediatrics journals. An Pediatr (Barc); 2015.
134. Barać L. Definitions and research of authorship across various scholarly disciplines : doctoral thesis. Zadar: University of Zadar; 2015.
135. Glujovsky D, Boggino C, Riestra B, Coscia A, Sueldo CE, Ciapponi A. Quality of reporting in infertility journals. Fertility and sterility. 2015;103(1):236-41.
136. Hartemink AE. The use of soil classification in journal papers between 1975 and 2014. Geoderma Regional. 2015;5:127-39.
137. Horvat M, Mlinaric A, Omazic J, Supak-Smolcic V. An analysis of medical laboratory technology journals’ instructions for authors. Science and engineering ethics. 2015;22(4):1095-106.
138. Koch M, Riss P, Kölbl H, Umek W, Hanzal E. Disclosures, conflict of interest, and funding issues in urogynecology articles: a bibliometric study. International urogynecology journal. 2015;26(10):1503-7.
139. Probst P, Hüttner FJ, Klaiber U, Diener MK, Büchler MW, Knebel P. Thirty years of disclosure of conflict of interest in surgery journals. Surgery. 2015;157(4):627-33.
140. Roig F, Borrego A. Conflict of interest disclosure policies in clinically oriented Spanish biomedical journals. Revista Española de Documentación Científica. 2015;38(3).
141. Smith TA, Kulatilake P, Brown LJ, Wigley J, Hameed W, Shantikumar S. Do surgery journals insist on reporting by CONSORT and PRISMA? A follow-up survey of ‘instructions to authors&#x2019. Annals of Medicine and Surgery. 2015;4(1):17-21.
142. Song T-J, Leng H-F, Zhong LL, Wu T-X, Bian Z-X. CONSORT in China: past development and future direction. Trials. 2015;16(1):243.
143. Stojanovski J. Do Croatian open access journals support ethical research? Content analysis of instructions to authors. Biochemia medica: Biochemia medica. 2015;25(1):12-21.
144. Stojanovski J, editor Journals' Editorial Policies-An Analysis of the Instructions for Authors of Croatian Open Access Journals. The International Conference on Electronic Publishing (Elpub); 2015; Valetta, Malta: IOS Press BV.
145. Teixeira RK, Yamaki VN, Pontes RV, Brito MV, da Silva JA. Evaluation of Ethical in Instructions to Authors of Brazilian Surgical Journals. Arq Bras Cir Dig. 2015;28(4):247-9.
146. Tierney E, O’Rourke C, Fenton J. What is the role of ‘the letter to the editor’? European Archives of Oto-Rhino-Laryngology. 2015;272(9):2089-93.
147. Yang W, Zou Q. The ethical issues in instructions for authors of Chinese biomedical journals. Learn Publ. 2015;28(3):216-22.
148. da Silva JAT. In defense of the use of italic for latin binomial plant names. Polish Botanical Journal. 2016;61(1):1-6.
149. Hernández-Ruiz A. Antifraud Editorial Policy in Spanish and Latin American Scientific Publication: JCR Social Sciences Edition/La política editorial antifraude de las revistas científicas españolas e iberoamericanas del JCR en Ciencias Sociales. Comunicar (English edition). 2016;24(48):19-27.

150. Hua F, Walsh T, Glenny A-M, Worthington H. Surveys on reporting guideline usage in dental journals. Journal of dental research. 2016;95(11):1207-13.
151. Janakiram C, Porteri C. Ethical process reporting in Indian dental journals. Accountability in research. 2016;23(3):163-77.
152. Jia Z, Wu Y, Tang Y, Ji W, Li W, Zhao X, et al. Equal contributions and credit: an emerging trend in the characterization of authorship in major spine journals during a 10-year period. European Spine Journal. 2016;25(3):913-7.
153. Koch M, Riss P, Umek W, Hanzal E. The explicit mentioning of reporting guidelines in urogynecology journals in 2013: a bibliometric study. Neurourology and urodynamics. 2016;35(3):412-6.
154. Koch M, Riss P, Umek W, Hanzal E. CONSORT and the internal validity of randomized controlled trials in female pelvic medicine. Neurourology and urodynamics. 2016;35(7):826-30.
155. Lei S-Y, Dong Y-P, Zhu W-F, Li L-J. An emerging trend of equal authorship credit in major public health journals. SpringerPlus. 2016;5(1):1083.
156. Liu T-Y, Cai S-Y, Nie X-L, Lyu Y-Q, Peng X-X, Feng G-S. The content of statistical requirements for authors in biomedical research journals. Chinese medical journal. 2016;129(20):2491.
157. Ma B, Ke FY, Zheng EL, Yang ZX, Tang QN, Qi GQ. Endorsement of the CONSORT statement by Chinese journals of Traditional Chinese Medicine: a survey of journal editors and review of journals' instructions for authors. Acupunct Med. 2016;34(3):178-83.
158. Mishra AK, Parmar A, Kaloiya GS, Balhara YPS, editors. A Descriptive Analysis of Instructions to Authors For Statistical Reporting of Article in Addiction Medicine Journals. ANCIPS; 2016: Indian J Psychiatry.
159. Nedovic D, Panic N, Pastorino R, Ricciardi W, Boccia S. Evaluation of the endorsement of the STrengthening the REporting of Genetic Association studies (STREGA) statement on the reporting quality of published genetic association studies. Journal of epidemiology. 2016;26(8):399-404.
160. Pouwels KB, Widyakusuma NN, Groenwold RH, Hak E. Quality of reporting of confounding remained suboptimal after the STROBE guideline. Journal of clinical epidemiology. 2016;69:217-24.
161. Roberts EA, Troiano C, Spiegel JH. Standardization of guidelines for patient photograph deidentification. Annals of plastic surgery. 2016;76(6):611-4.
162. Shamseer L, Hopewell S, Altman DG, Moher D, Schulz KF. Update on the endorsement of CONSORT by high impact factor journals: a survey of journal “Instructions to Authors” in 2014. Trials. 2016;17(1):301.
163. Sims MT, Henning NM, Wayant CC, Vassar M. Do emergency medicine journals promote trial registration and adherence to reporting guidelines? A survey of “Instructions for Authors”. Scandinavian journal of trauma, resuscitation and emergency medicine. 2016;24(1):137.
164. Vetter D, Storch I, Bissonette JA. Advancing landscape ecology as a science: the need for consistent reporting guidelines. Landscape ecology. 2016;31(3):469-79.
165. Bhat A, Shah A, Sherighar SG. Instructions to prospective authors by Indian biomedical journals: An opportunity to promote responsible conduct of research. Journal of Empirical Research on Human Research Ethics. 2017;12(2):117-23.
166. Bolshete P. Authorship criteria and reporting of ethical compliance in Indian biomedical journals. Indian journal of medical ethics. 2017;2(3):160-4.
167. Martin G, Clarke RM. Are psychology journals anti-replication? A snapshot of editorial practices. Frontiers in psychology. 2017;8:523.
168. Mercieca-Bebber R, Rouette J, Calvert M, King MT, McLeod L, Holch P, et al. Preliminary evidence on the uptake, use and benefits of the CONSORT-PRO extension. Quality of Life Research. 2017;26(6):1427-37.
169. Pieper D, Mathes T. Survey of instructions for authors on how to report an update of a systematic review: guidance is needed. Evidence-based medicine. 2017;22(2):45-8.
170. Shamseer L, Moher D, Maduekwe O, Turner L, Barbour V, Burch R, et al. Potential predatory and legitimate biomedical journals: can you tell the difference? A cross-sectional comparison. BMC medicine. 2017;15(1):28.

171. Tam WW, Lo KK, Khalechelvam P. Endorsement of PRISMA statement and quality of systematic reviews and meta-analyses published in nursing journals: a cross-sectional study. BMJ open. 2017;7(2):e013905.
172. Taquette SR, Villela WV. Knowledge references: analysis of Brazilian health journal instructions to authors. Ciencia & saude coletiva. 2017;22(1):7-13.
173. Wayant C, Smith C, Sims M, Vassar M. Hematology journals do not sufficiently adhere to reporting guidelines: a systematic review. Journal of Thrombosis and Haemostasis. 2017;15(4):608-17.
174. ICMJE. Recommendations for the Conduct, Reporting, Editing, and Publication of Scholarly work in Medical Journals 2017 [Available from: http://www.icmje.org/recommendations/.
